# Discovery of dual rho-associated protein kinase 1 (ROCK1)/apoptosis signal–regulating kinase 1 (ASK1) inhibitors as a novel approach for non-alcoholic steatohepatitis (NASH) treatment

**DOI:** 10.1186/s13065-023-01081-3

**Published:** 2024-01-03

**Authors:** Yara A. Zaky, Mai W. Rashad, Marwa A. Zaater, Ahmed M. El Kerdawy

**Affiliations:** 1 Department of Chemistry, School of Pharmacy, Newgiza University (NGU), Newgiza, Km 22 Cairo-Alexandria Desert Road, Cairo, Egypt; 2 Master Postgraduate Program, Department of Pharmaceutical Chemistry, Faculty of Pharmacy, Cairo University, Cairo, Egypt; 3 Department of Pharmaceutical Chemistry, Faculty of Pharmacy, Cairo University, Cairo, Egypt; 4 School of Pharmacy, College of Science, University of Lincoln, Joseph Banks Laboratories, Green Lane, Lincoln, Lincolnshire UK

**Keywords:** NASH, ASK1, MAP3K5, ROCK1, Pharmacophore modeling, Molecular docking, Virtual screening, Kinase inhibitor

## Abstract

In the current study we suggest a novel approach to curb non-alcoholic steatohepatitis (NASH) progression, and we suggest privileged scaffolds for the design of novel compounds for this aim. NASH is an advanced form of non-alcoholic fatty liver disease that can further progress into fibrosis, cirrhosis, and hepatocellular carcinoma. It is a widely emerging disease affecting 25% of the global population and has no current approved treatments. Protein kinases are key regulators of cellular pathways, of which, Rho-associated protein kinase 1 (ROCK1) and apoptosis signal–regulating kinase 1 (ASK1) play an important role in the progression of NASH and they stand out as promising targets for NASH therapy. Interestingly, their kinase domains are found to be similar in sequence and topology; therefore, dual inhibition of ROCK1 and ASK1 is expected to be amenable and could achieve a more favourable outcome. To reach this goal, a training set of ROCK1 and ASK1 protein structures co-crystalized with type 1 (ATP-competitive) inhibitors was constructed to manually generate receptor-based pharmacophore models representing ROCK1 and ASK1 inhibitors’ common pharmacophoric features. The models produced were assessed using a test set of both ROCK1 and ASK1 actives and decoys, and their performance was evaluated using different assessment metrics. The best pharmacophore model obtained, showing a Mathew’s correlation coefficient (MCC) of 0.71, was then used to screen the ZINC purchasable database retrieving 6178 hits that were filtered accordingly using several medicinal chemistry and pharmacokinetics filters returning 407 promising compounds. To confirm that these compounds are capable of binding to the target kinases, they were subjected to molecular docking simulations at both protein structures. The results were then assessed individually and filtered, setting the spotlight on various privileged scaffolds that could be exploited as the nucleus for designing novel ROCK1/ASK1 dual inhibitors.

**Supplementary Information:**

The online version contains supplementary material available at 10.1186/s13065-023-01081-3.

## Introduction

It is estimated that around one-quarter of the global population has non-alcoholic fatty liver disease (NAFLD) [1]. It is the fastest-growing cause of hepatocellular carcinoma in the United States, France, and the United Kingdom and affects 47.5% of the Egyptian population as well [2, 3]. NAFLD is a benign condition characterized by the build-up of lipids in hepatocytes and affects at least 5% of the liver volume, which can progress into non-alcoholic steatohepatitis (NASH), an advanced form of NAFLD [4, 5]. NASH is characterized by steatosis and inflammation of the hepatocytes; therefore, it can progress further into fibrosis, cirrhosis, liver cancer, and even death owing to the persistent hepatocytes’ inflammation and long-term damage [5–8]. In Egypt alone, approximately 56.7% of NAFLD patients have liver fibrosis [3]. Overall, 20% of NAFLD patients are likely to progress into NASH patients increasing the health and economic burden further [9].

Currently, there is not any approved medication available for the treatment of NAFLD or NASH [4]. As for NASH, physicians usually recommend weight loss and/or bariatric surgeries [10–12]. Alternatively, there are several potential pharmacological strategies for reducing hepatic steatosis including fatty acid synthase inhibitors, ketohexokinase inhibitors, and sodium-glucose co-transporter 2 inhibitors [13].

Protein kinases (PKs) play a vital role in the progression of NASH [14]. In particular, Rho-Associated Protein Kinase 1 (ROCK1) and Apoptosis Signal–Regulating Kinase 1 (ASK1) (MAP3K5) are found to play an important role in the progression of NAFLD to NASH through mediating lipotoxic effects [15]. This makes them promising drug targets for NASH treatment [14].

The kinase catalytic domain can be divided into two main lobes; a smaller N-terminal lobe and a larger C-terminal lobe which are connected by a hinge region [16]. ATP binds in the cleft formed by the folding of the large and the small lobes [16, 17]. Both lobes are found to be conserved between different protein kinases [18]. The N-terminal lobe is formed of 5 β-sheets and 1 α-helix (α-C helix) that is located at the end of the lobe connecting it to the hinge region [16, 17]. The α-C helix can be found in two conformations, in or out, making the protein kinase either active or inactive, respectively [16, 17]. On the other hand, the C-terminal lobe is formed of 8 α-helices and 4 conserved short β sheets [16, 17]. The C-lobe contains the DFG motif, which can be present in an active or inactive conformation, either in or out, respectively [19]. When found in the active conformation, the aspartate of the DFG motif binds to Mg^2+^ (or Mn^2+^) to coordinate and facilitate ATP binding [20].

There are six different types of PK inhibitors (I–VI) which are divided according to their position of binding at the protein kinase, their interaction type with the protein kinase (Covalent or non-covalent), and the conformation adopted by the protein kinase upon binding [21–24]. Type I inhibitors, which will be the focus of this study, bind in a competitive mode to the ATP binding site of the protein kinase active conformation with both DFG and α-C helix adopting the “in” conformation [22, 23, 25]. To achieve better selectivity towards the target kinase, type I inhibitors extend to further regions such as the front pocket, DFG-motif, gate area, and the p-loop region [26].

ROCK1, a serine/threonine kinase member of the ACG kinase family, is strongly linked to NASH as it regulates several processes involved in NASH pathogenesis [15, 27]. Upon its activation, ROCK1 stimulates lipogenic pathways, which presents direct evidence for its involvement in NASH progression along with its induction of insulin resistance [15, 28]. ROCK1 is involved in mediating hepatocyte lipotoxic signalling as well [15]. Moreover, the apparent activation of ROCK1 in individuals on a high-fat diet is an additional proof on ROCK1 involvement in lipogenic pathways [28].

**Fig. 1:**
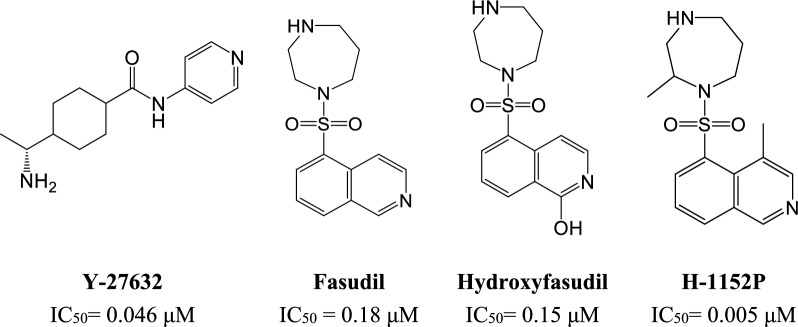
Structures of some reported ROCK1 inhibitors with their ROCK1 IC_50_ values [29, 37–39]

Y-27632 (Fig. 1) is a promising selective ROCK1 inhibitor with an IC_50_ value of 0.046 μM that restrain the progression of liver steatosis and fibrosis in NASH rat model [29]. Fasudil (Fig. 1), a dual ROCK1/2 inhibitor approved in Japan and China for cerebral vasospasm and pulmonary hypertension, is a promising agent for NASH treatment due to its observed safety, efficacy, and anti-fibrotic effect (IC_50_ = 0.18 μM [ROCK1] and 0.06 μM [ROCK2]) [30]. When tested on NASH animal models, fasudil reduced liver injury, macrophage-associated inflammation, and fibrosis [31]. The first identified fasudil metabolite, hydroxyfasudil (Fig. 1), is another potential ROCK1 inhibitor which is more selective towards ROCK1 than its parent compound (IC_50_ = 0.15 μM [ROCK1] and 0.57 μM [ROCK2]) [32, 33]. On the other hand, the dimethylated fasudil analogue, H-1152P (Fig. 1), is a more potent ROCK1 inhibitor than the previously mentioned derivatives with IC_50_ value of 0.005 μM [34].

In ROCK1 kinase domain, Y-27632 interacts with the key amino acids Met156 (hinge region), Asp216 (DFG-motif), Glu154 (αC helix), and Asn203 which coordinates the Mg^2+^ cation with Asp216 during catalysis [33]. Generally, ROCK1 ATP-competitive inhibitors interact through hydrogen bonding at the hinge region with Tyr155 and Met156 and at the catalytic loop with Lys105 [35, 36]. Furthermore, the compounds’ aromatic core is sandwiched between Val90 and Leu205 [35].

On the other hand, ASK1 (MAP3K5) is a serine/threonine kinase member of the mitogen-activated protein kinase (MAPK) family [40]. Under lipotoxic stress conditions, ASK1 activation leads to insulin resistance, apoptosis, lipogenesis, and hepatic stellate cells activation inducing hepatic fibrosis through the release of proinflammatory and profibrotic factors [41].

**Fig. 2:**
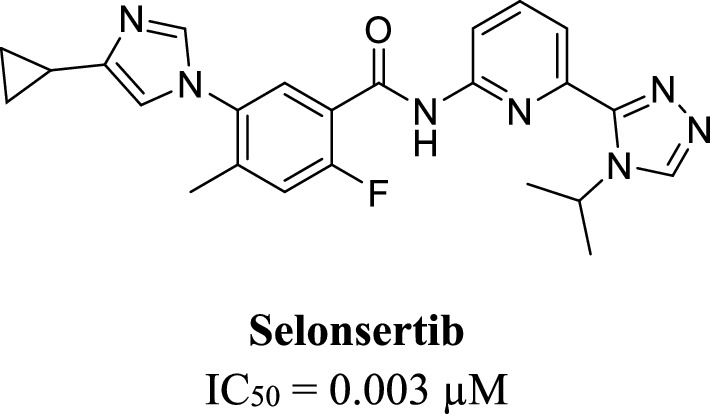
The structure of selonsertib, a potent ASK1 inhibitor [42, 49]

Selonsertib (Fig. 2), discovered by Gilead Sciences, is the first and only reported ASK1 inhibitor progressed to the clinical phase [42]. In one study, selonsertib was found to be safe, effective, and reduced liver fibrosis in patients [43]. However, in another study, selonsertib showed a lack of efficacy, which in turn lead to the termination of its clinical testing [44]. Selonsertib showed a potent ASK1 inhibitory activity with IC_50_ value of 0.003 µM [42]. In ASK1 kinase domain, selonsertib interacts with the key amino acids Lys709 and Val757 through multiple hydrogen bonds [45]. The removal of its 4-isopropyl-1,2,4-triazole moiety reduced its potency by a 1000-fold due to the loss of the hydrogen bond interaction with Lys709 [45]. Generally, ASK1 ATP-competitive inhibitors interact through hydrogen bonding at the hinge region with Gln756 and Val757 and at the catalytic loop with Lys709 [46, 47]. Furthermore, the compounds’ aromatic core is surrounded by Val694 and Leu810 [48].

In this context, to date, inhibitors that are tested for NASH are either ROCK1 specific inhibitors or ASK1 specific ones. However, multi-kinase inhibition has several benefits, such as increasing potency due to its synergistic effect, reducing the possibility of polypharmacy toxicity, bypass pharmacokinetics incompatibilities, and enhancing selectivity [50, 51].

Inspired by the previously mentioned facts, the current study aims to discover dual ROCK1/ASK1 inhibitors as a novel approach to efficiently hinder NASH progression. To this end, the Protein Data Bank (PDB) (https://www.rcsb.org/) will be used to generate a training set of ROCK1 and ASK1 protein structures bound to type I inhibitors. Receptor-based pharmacophore models will be then manually generated based on the common interactions extracted from the co-crystalized inhibitors in both proteins. The generated pharmacophore models will be filtered and validated according to a compiled test set of ROCK1/ASK1 active inhibitors and inactive decoys. The pharmacophore model survives the filtration and validation step will be then used to screen the ZINC purchasable database [52]. Hits retrieved from the virtual screening process will be studied individually and filtered to keep only promising lead-like compounds with acceptable pharmacokinetic properties. Molecules survive the filtration step will be then subjected to molecular docking simulations in ROCK1 and ASK1 kinase domains. Molecules’ docking poses will be then evaluated to extract molecules that bind similarly in both proteins performing the essential interactions. This is followed by representative molecular dynamics simulations to study the stability of the obtained molecular docking poses. Finally, molecules with promising docking poses in both proteins will be clustered to identify privileged scaffolds for dual ROCK1/ASK1 inhibition.

## Results and discussion

To validate the rational of our novel approach, the similarity of ROCK1 and ASK1 kinase domains in sequence, topology, and structure was initially investigated. The amino acid sequences of ROCK1 and ASK1 kinase domains were first obtained from UniProt (https://www.uniprot.org) (UniProt ID: Q13464 and Q99683, respectively). Then, both kinase domains’ amino acid sequences were aligned using NCBI Basic Local Alignment Search Tool (BLAST) for proteins (BLASTp) (https://blast.ncbi.nlm.nih.gov/Blast.cgi). A sequence similarity of 32.37% was found between both kinase domains with a percentage of positives of 52%, indicating that most differences in sequence were due to conservative substitutions.

**Fig. 3:**
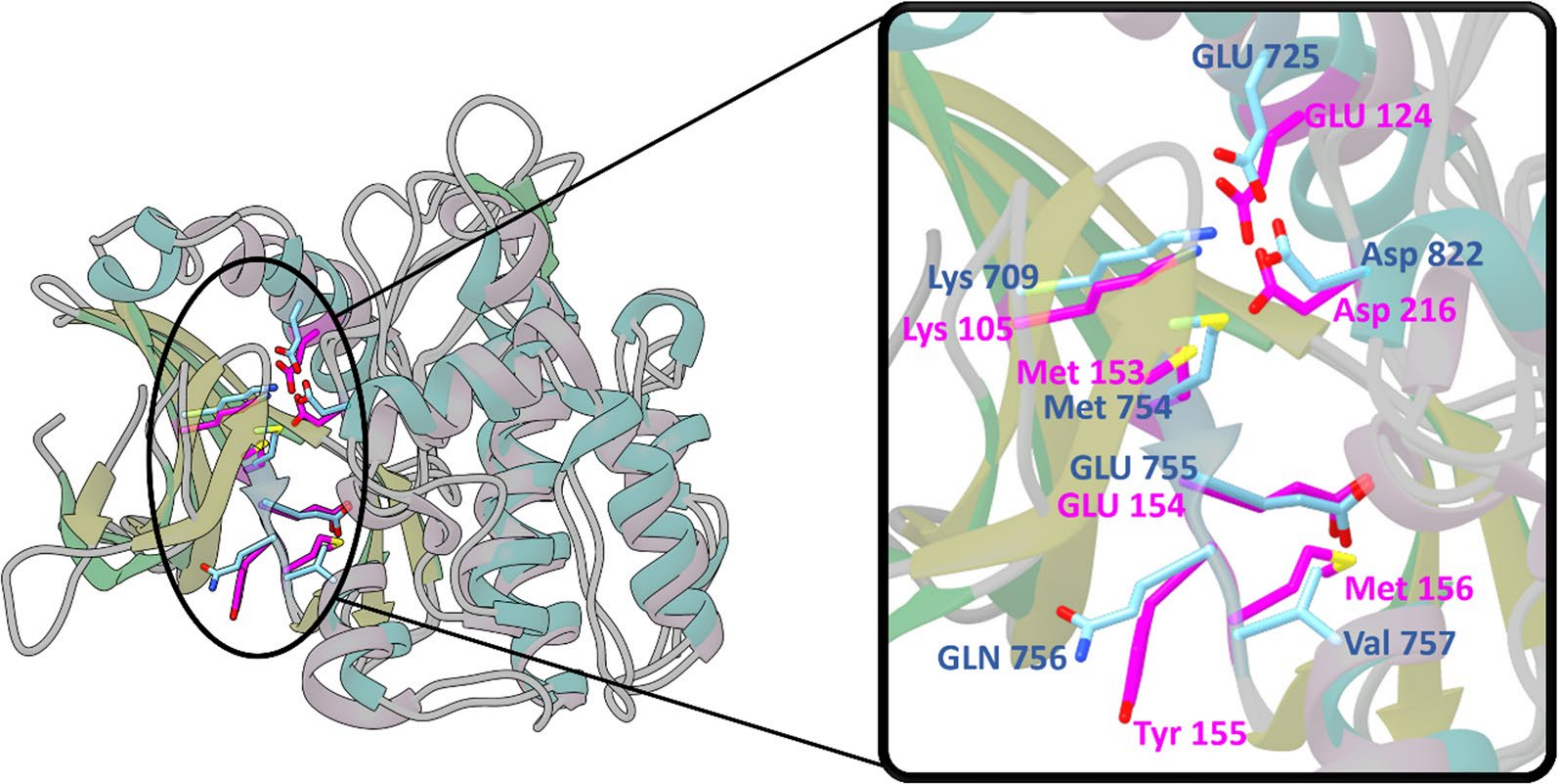
3D superimposition of ROCK 1 (PDB ID: 4YVC) and ASK1 (PDB ID: 5V24) protein structures with a close focus on the key residues at the kinase domain (4YVC residues: light blue, 5V24 residues: magenta)

Furthermore, two crystal structures of ROCK1 and ASK1 (PDB ID: 4YVC and 5V24, respectively) were downloaded from the protein data bank (PDB) (https://www.rcsb.org/). Then, they were aligned, and their superposition quality was investigated, especially, the ATP-binding site and its key amino acids (Fig. 3 and Table 1). The obtained superposition exhibited an overall RMSD value of 2.80Å indicating that the protein structures are well aligned (Fig. 3). In addition, the key residues at the ATP-binding site had positional RMSD values’ range of 0.62–1.57Å (Table 1). These results reflect the high topological similarity shared between the two proteins’ kinase domains, especially, within the ATP-binding site.

**Table 1:** The superposition of the key amino acid residues involved in type I ATP-competitive inhibitor binding in ROCK1 and ASK1 and their corresponding positional RMSD values

ROCK1 Residue	ASK1 Residue	RMSD value (Å)
Lys105	Lys709	1.321
Met153	Met754	1.559
Glu154	Glu755	1.576
Tyr155	Gln756	1.511
Met156	Val757	0.866
Asp216	Asp822	0.626

Table 1 and Fig. 3 show that the key binding residues are not only showing high similarity in their position within the binding pocket in 3D space but also in their type and nature. The hinge region residues, Met153, Glu154, Tyr155, Met156 in ROCK1 align with Met754, Glu755, Gln756, Val757 in ASK1, respectively. Tyr155 (ROCK1) and Gln756 (ASK1) differ in nature, but Met156 (ROCK1) and Val757 (ASK1) share the same hydrophobic nature. Catalytic residues Lys105 (ROCK1) and Lys709 (ASK1), within the catalytic loop, also overlay in 3D space. The αC helix Glu124 (ROCK1) and Glu725 (ASK1) residues are also found to converge in 3D space. Thus, it is obvious that the interesting regions for drug discovery such as the hinge region and catalytic loop align and show high residue similarity.

These preliminary studies indicated that ROCK1 and ASK1 are not only related to NASH pathophysiology and progression but also with similar kinase domains in sequence, topology, and structure and so amenable to dual inhibition. Thus, designed dual ROCK1/ASK1 inhibitors could effectively target the kinase domains of both proteins.

### Selection of the X-ray crystallographic structures for ROCK1 and ASK1 and training set generation

There are many X-ray crystal structures for both ROCK1 and ASK1 available in the PDB and all were retrieved. All crystal structures were wild type protein structures bound to type I ATP-competitive kinase inhibitors which bind to the kinases’ active conformation with both DFG and αC-helix are adopting the “in” conformation (DFG-in and αC-helix-in) [22, 23]. There were twenty-three ROCK1 and nineteen ASK1 extracted crystal structures co-crystallized with various type I inhibitors.

To design dual ROCK1/ASK1 inhibitors, a selected group of protein structures were chosen according to their similarities in the inhibitors’ binding pattern and the region of binding in the kinase domain. This yielded a group of three ROCK1 (PDB ID: 7JOU, 4YVC, and 4W7P) and four ASK1 (PDB ID: 6VRE, 5V24, 5VIL, and 5UOX) crystal structures forming the training set. The inhibitor molecules in the chosen structures were bound to the proteins at the hinge region and the catalytic loop via hydrogen bonding (Tables 2 and 3). They interact at the hinge region with Tyr155 & Met156 (ROCK1) and Gln756 & Val757 (ASK1) and at the catalytic loop with Lys105 (ROCK1) and Lys709 (ASK1) (See Additional file 1: S1 for further details).

**Table 2:** ROCK1 training set

PDB ID	Ref	Ligand structure	Ligand/ROCK1 interactions
7JOU	[53]		
4YVC	[54]		
4W7P	[55]

**Table 3:** ASK1 training set

PDB ID	Ref	Ligand structure	Ligand/ASK1 interactions
6VRE	[56]		
5V24	[45]		
5VIL	[46]		
5UOX	[47]		

### Test set generation

Two separate test sets were compiled one for each protein. Regarding ROCK1, a pre-compiled test set was available on DEKOIS 2.0 (http://www.pharmchem.uni-tuebingen.de/dekois/) [57]. As for ASK1, there was not any pre-compiled test set available in the searched databases, thus, a set of active compounds was self-collected from the PDB and several additional research papers [46, 47, 56, 58–60]. The decoys were then generated using DUD-E (http://dude.docking.org/generate) [61]. The compiled actives and inactives were then subjected to conformational search. The final compiled test set contains 98,239 conformations of 39 active compounds (19 ROCK1 inhibitors and 20 ASK1 inhibitors) (Tables 4 and 5) and 1198 decoys (598 for ROCK1 inhibitors and 600 for ASK1 inhibitors). Active/inactive ratio was maintained (> 1:30) to mimic that in the natural chemical space [62].

**Table 4:**
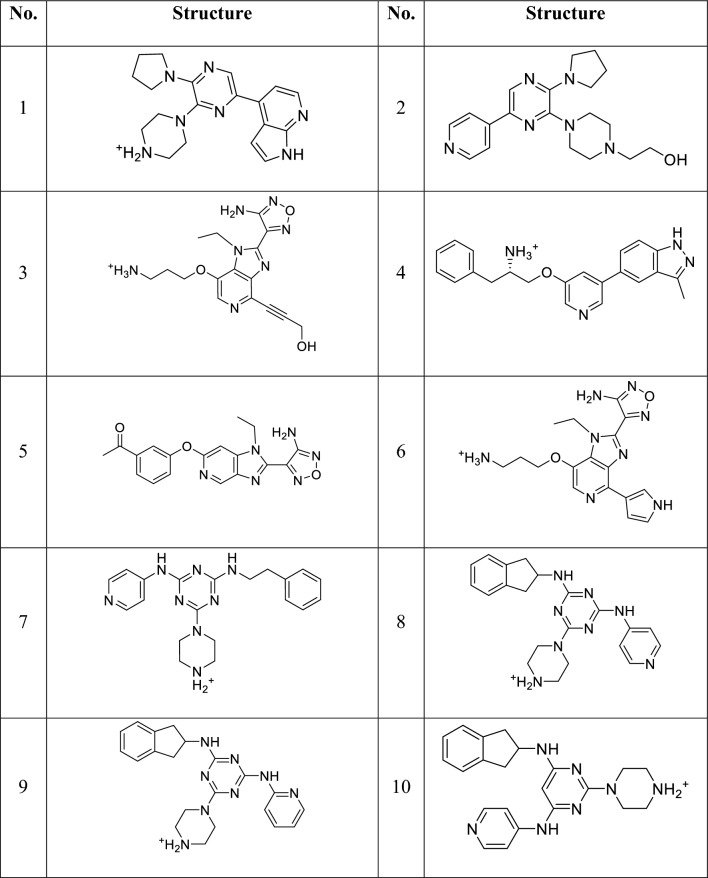

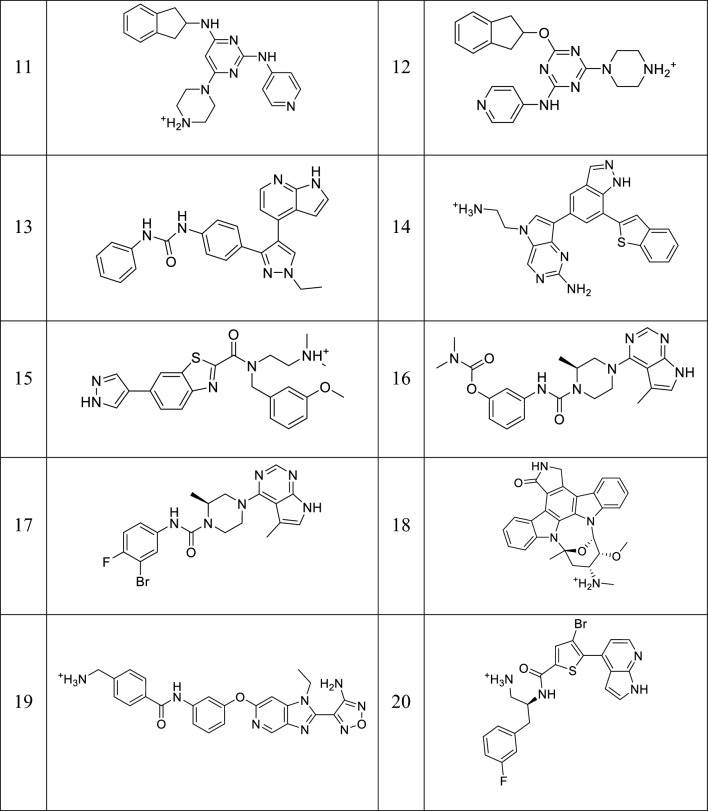
The active compounds in ROCK1 test set

**Table 5:** The active compounds in the Self-collected ASK1 test set

No	Structure	Ref	No	Structure	Ref
1		[49]	2		[59]
3		[59]	4		[58]
5		[46]	6		[47]
7		[46]	8		[47]
9		[47]	10		[46]
11		[63]	12		[56]
13		[60]	14		[46]
15		[46]	16		[46]
17		[64]	18		[46]
19		[58]	20		[47]

### Pharmacophore models generation

To obtain a common pharmacophore model describing the shared key features in the inhibitors of both proteins, different pharmacophore models were manually generated using receptor-based pharmacophore modeling. This was carried out by initially aligning the seven prepared protein structures of the training set X-ray crystal structures (Tables 2 and 3).

After visually inspecting the various aligned protein structures and noting the common ligands’ interactions within the kinase domains, various qualitatively and quantitively different 3D pharmacophore models were manually generated. The constructed pharmacophore models were different in the features selected in terms of their number and location, the radii of the individual features, and the excluded volumes used. Excluded volumes were important in defining the binding pocket and the steric extent of the amino acid residues lining the binding site. In some of the constructed pharmacophore models, excluded volumes were automatically constructed based on the ROCK1 (PDB ID: 4W7P) binding site. PDB ID: 4W7P’s pocket was chosen due to its large size reflected by binding to the largest inhibitor of the training set, thereby making the excluded volumes less restrictive. Overall, thirteen receptor-based pharmacophore models were generated (See Additional file 1: S3 for further details).

### Pharmacophore selection and validation

Pharmacophore models, as a virtual screening protocol, are commonly assessed based on their ability to discriminate between active and inactive compounds. Pharmacophore models performing efficiently and enriching the selected hit set with active compounds are progressed further to be used in virtual screening. In the current work, the performance of the generated pharmacophore models was evaluated using the compiled test set.

Different assessment metrics were used to assess the performance of the constructed pharmacophore models on the compiled test set to select the best-performing model. Those metrics included sensitivity (Se), specificity (Sp), yield of actives (Ya), enrichment (E), accuracy (Acc), discrimination ratio (DR), F_1_ score (F_1_), and Mathew’s correlation coefficient (MCC) (see Additional file 1: S2 for further details). Table 6 reports the results of each of the constructed pharmacophore models.

**Table 6:** Pharmacophore models’ performance on the test set (Selected model metrics are shown in **Bold**)

MODEL No	n	TP (ROCK1 + ASK1)*	FP	FN	TN	Se	Sp	ya	e	Acc	DR	f_1_	MCC
1	644	36 (16 + 20)	608	3	590	0.923	0.492	0.056	1.773	0.506	1.874	0.105	0.145
2	744	36 (16 + 20)	708	3	490	0.923	0.409	0.048	1.535	0.425	2.257	0.092	0.119
3	172	32 (13 + 19)	140	7	1058	0.821	0.883	0.186	5.901	0.881	0.929	0.303	0.355
4	352	30 (11 + 19)	322	9	876	0.769	0.731	0.085	2.703	0.732	1.052	0.154	0.194
5	302	26 (8 + 18)	277	13	921	0.667	0.769	0.086	2.722	0.766	0.867	0.1520	0.177
6	191	18 (7 + 11)	172	21	1026	0.462	0.856	0.095	3.005	0.844	0.539	0.1572	0.154
7	364	29 (11 + 18)	336	10	862	0.744	0.720	0.079	2.520	0.720	1.033	0.1436	0.177
8	625	36 (16 + 20)	589	3	609	0.923	0.508	0.058	1.827	0.521	1.816	0.108	0.151
9	129	36 (16 + 20)	94	3	1104	0.923	0.922	0.277	8.783	0.922	1.002	0.4260	0.481
10	399	34 (14 + 20)	365	5	833	0.872	0.695	0.085	2.703	0.701	1.254	0.155	0.212
11	**57**	**34 (14 + 20)**	**23**	**5**	**1175**	**0.872**	**0.981**	**0.596**	**18.919**	**0.977**	**0.889**	**0.708**	**0.711**
12	67	35 (15 + 20)	32	4	1166	0.897	0.973	0.522	16.569	0.971	0.922	0.660	0.672
13	77	36 (17 + 19)	40	3	1158	0.923	0.967	0.474	15.024	0.965	0.955	0.6261	0.647

**n** is the number of hits selected by the pharmacophore model, **TP** is the number of true positive, **FP** is the number of false positive, **FN** is the number of false negative, **TN** is the number of true negative, **Se** is sensitivity, **Sp** is specificity, **Ya** is yield of actives,** E** is enrichment**, Acc** is accuracy, **DR** is discrimination ratio,** F**_**1**_is F_1_ score, and **MCC** is Mathew’s correlation coefficient. *Number of ROCK1 TP + number of ASK1 TP

Generally, pharmacophore models that had few or no excluded volumes performed worse than the rest of the models. Moreover, despite their ability to detect many active compounds, pharmacophore models with large-radii features lacked specificity as they detected many false positives as well. Overall, the qualitative and quantitative diversity among the different pharmacophore models was important to be able to reach the optimal model which can accurately detect both ROCK1 and ASK1 active compounds while filtering out the inactive decoys (See Additional file 1: S3 for further details about the features in each pharmacophore model).

**Fig. 4:**
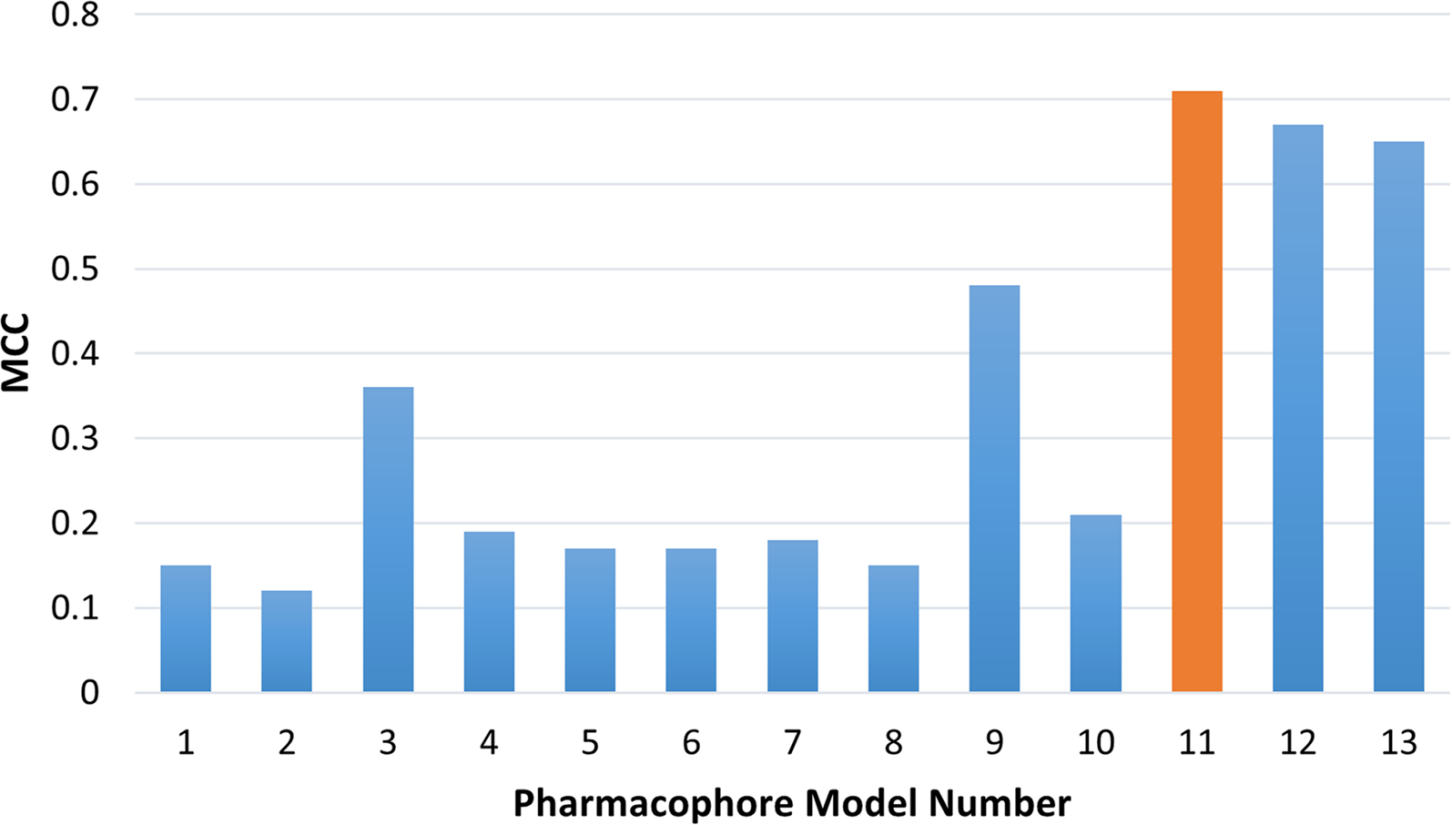
The overall performance of the different pharmacophore models based on their Mathew’s correlation coefficient (MCC) values

As can be seen in Table 6, pharmacophore models 1, 2, and 8 exhibited good sensitivity, with a value of 0.923, meaning that they yielded a high number of true positives; however, they showed low specificity (0.492, 0.409, and 0.508, respectively) as they could not discard decoys properly and regarded them as hits, FP (608, 708, and 589); so, these models were biased towards active compounds, and this is reflected in their low MCC (0.145, 0.119, and 0.151, respectively) (Table 6 and Fig. 4). This could be attributed to using fewer excluded volumes, fewer pharmacophoric features, and/or large-radii features in these models.

On the contrary, pharmacophore models 5,6, and 7 showed low sensitivity (0.667, 0.462, and 0.744, respectively), meaning that they yielded a low number of true positives TP (26, 18, and 29, respectively); on the other hand, they showed reasonable specificity (0.769, 0.856 and 0.720, respectively) so, could discard decoys and correctly consider them as inactive compounds. Therefore, these models were biased towards inactive compounds, and this is also reflected in their low MCC (0.177, 0.154, and 0.177, respectively) (Table 6 and Fig. 4).

Pharmacophore models 11, 12, and 13 showed a balance between sensitivity and specificity with a sensitivity of 0.872, 0.897, and 0.923, respectively, and a specificity of 0.981, 0.973, and 0.967, respectively. This indicates that these models are not biased towards either of actives or decoys and can classify them correctly, and this is reflected in their high MCC values (0.711, 0.672, and 0.647, respectively) (Table 6 and Fig. 4). Figure 4 compares the performance of the different pharmacophore models using their corresponding MCC values.

### Selected 3D pharmacophore model

It is important for the selected pharmacophore model to be able to represent the key binding features for both ROCK1 and ASK1 in the correct location and be with appropriate radii. Pharmacophore model 11 was the best at describing molecular features required for essential interactions in both proteins. Thus, it exhibited the best performance among all the pharmacophore models with the highest F_1_ score and MCC values (0.708 and 0.711, respectively).

As depicted in Table 6, pharmacophore 11 has a sensitivity (*Se*) of 0.872, detecting 34 out of 39 actives. Moreover, it discarded 1175 out of 1198 decoys, having a specificity (*Sp*) of 0.981. Values of sensitivity and specificity indicated that this pharmacophore model has a good ability to detect actives and disregard decoys. It had a yield of actives (*Ya*) of 59.6%, detecting a total of 57 compounds as hits, 34 of which are active. In addition, it had an enrichment value (*E*) of 18.91, proving the ability of the pharmacophore model to perform better than random screening. Furthermore, it had an accuracy (*Acc*) of 0.977 highlighting that this model can identify active compounds and eliminate decoys. Finally, this pharmacophore model had a discrimination ratio (DR) of 0.899 emphasising its ability to differentiate between active and inactive compounds.

**Fig. 5:**
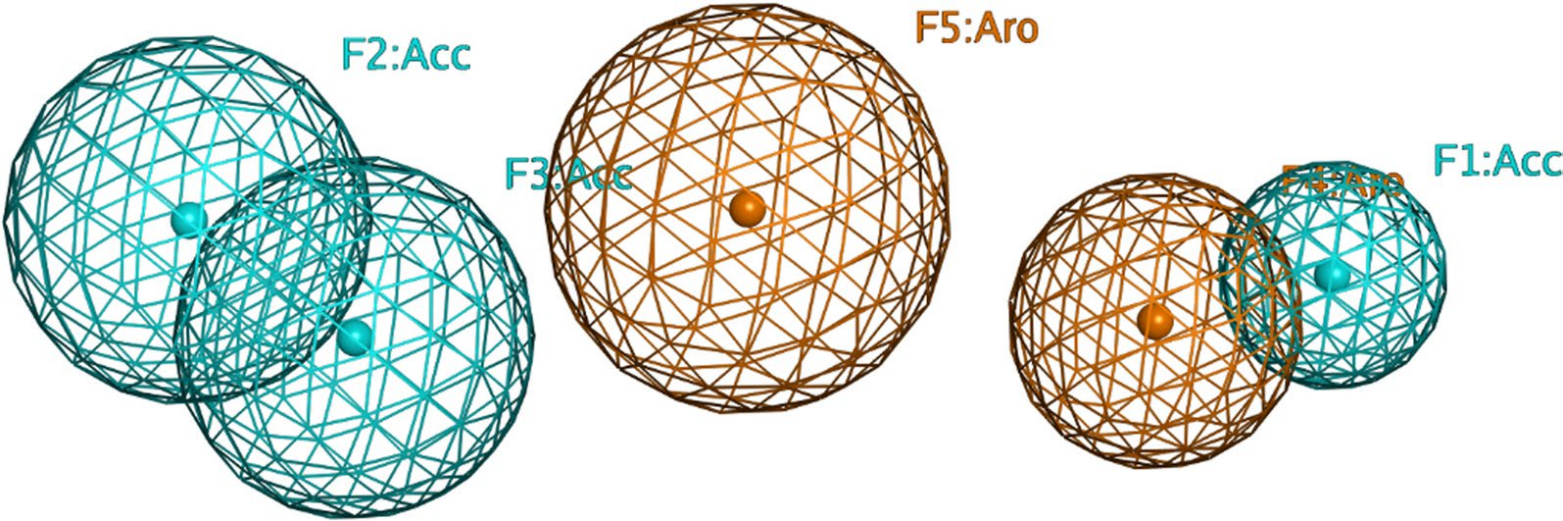
The selected 3D pharmacophore model (Model 11)

Figure 5 portrays the selected pharmacophore model (Model 11) showing the selected features in the 3D space. First feature (F1:Acc) represents a hydrogen bond acceptor for the interaction with the ROCK1/ASK1 catalytic Lys105/709. Second and third features (F2:Acc and F3:Acc) represent two constrained features required for the interaction with ROCK1/ASK1 at the hinge region with Tyr155 & Met156 and Glu756 & Val757, respectively. Finally, features (F4:Aro and F5:Aro) represent the aromatic features located at the centre of the model near the ROCK1/ASK1 catalytic domain in proximity to Val90/694. Features F1, F4, and F5 were defined essential, while F2 and F3 were partial features with a minimum requirement of total four features. Automatic and manual excluded volumes were added using the binding pocket of ROCK1 (PDB ID: 4W7P) to define the binding site steric extent. Table 7 shows the Inter-feature distance matrix for the selected pharmacophore model (Model 11).

**Table 7:** Inter-feature distance matrix for the selected pharmacophore model (Model 11) (Distances are in Å)

	F1:Acc	F2:Acc	F3:Acc	F4:Aro	F5:Aro
F1:Acc	0				
F2:Acc	10.07	0			
F3:Acc	8.67	1.84	0		
F4:Aro	1.61	8.55	7.10	0	
F5:Aro	5.20	4.90	3.68	3.74	0

**Fig. 6:**
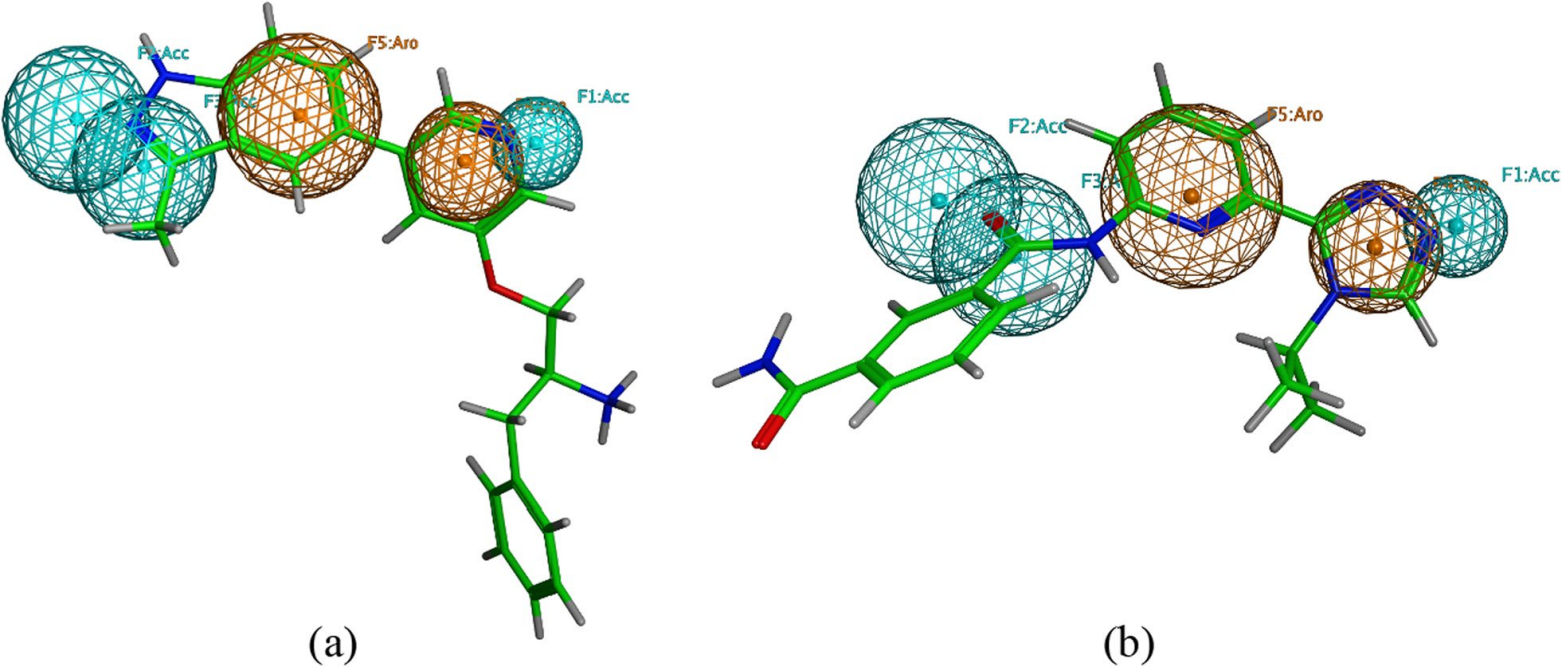
The selected pharmacophore model (Model 11) mapped onto the active compound **4** from ROCK1 test set (**a**) and compound **1** from ASK1 test set (**b**). Both compounds exhibited the lowest RMSD values amongst the other test set active compounds

Figure 6 shows pharmacophore model 11 mapped onto two representative active compounds from the compiled test set. Compound **4** of ROCK1 test set exhibited the lowest RMSD value of 0.749Å. This indicates the good alignment of the molecule’s structural features with the pharmacophoric features of the selected pharmacophore (Fig. 6a). The nitrogen of the pyridine ring is mapped onto the acceptor feature (F1:Acc) of the pharmacophore model. The pyridine ring also aligns with the aromatic feature (F4:Aro). The benzene ring of the indazole aligns with the aromatic feature (F5:Aro). Finally, one nitrogen of the indazole ring is mapped onto the acceptor feature (F2:Acc). Alternatively, compound **1** of ASK1 test set exhibited the lowest RMSD score of 0.611Å, indicating its low deviation from the pharmacophoric feature centres (Fig. 6b). One nitrogen of the triazole ring aligns with the acceptor feature (F1:Acc). The triazole ring aligns with the aromatic feature (F4:Aro) as well. The pyridine ring aligns with the aromatic feature (F5:Aro). Whereas the oxygen of the amide carbonyl group aligns with the acceptor feature (F3:Acc).

### Virtual screening and hit filtration

**Fig. 7:**
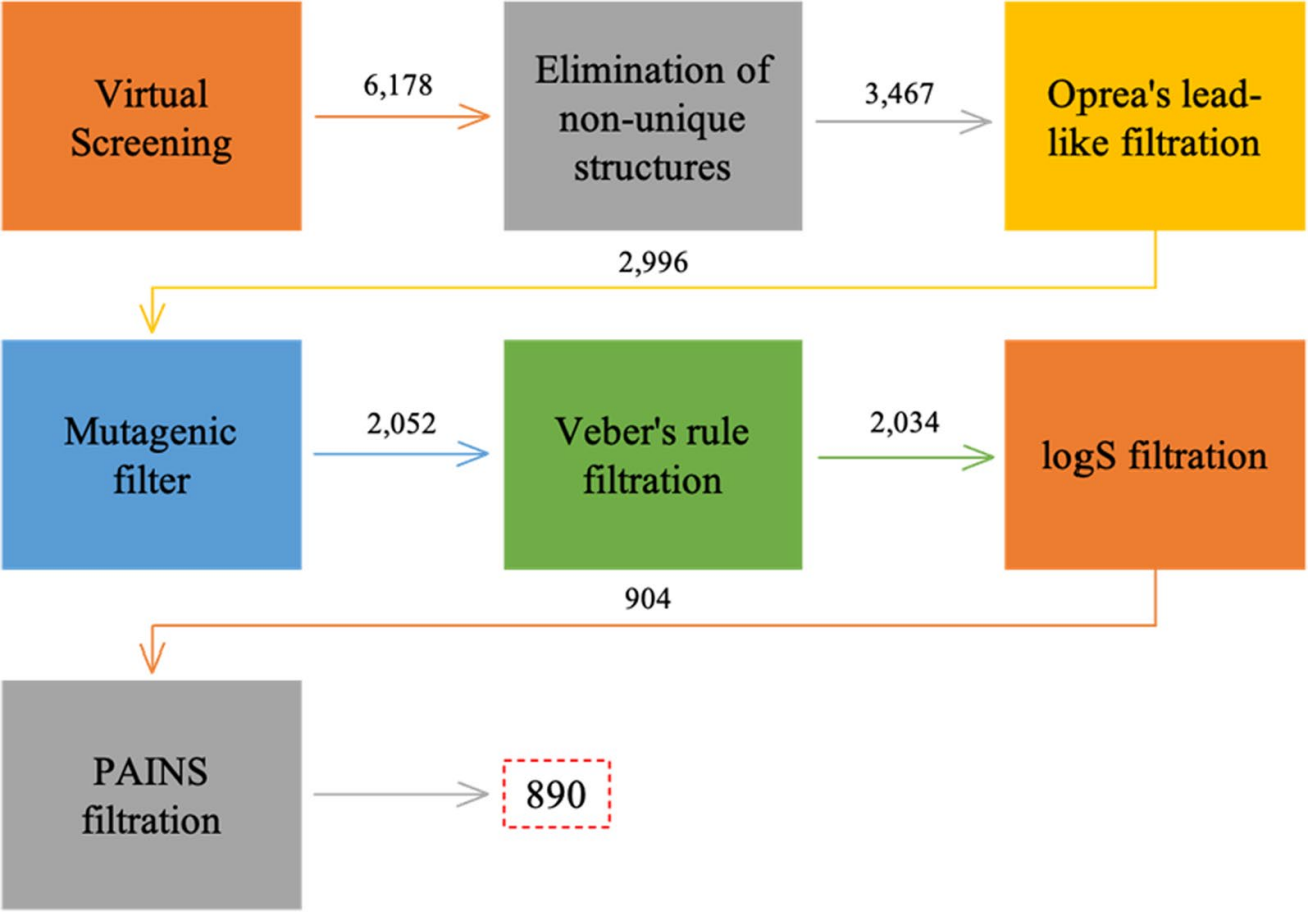
Virtual screening and hit filtration process

Virtual screening was done using the ZINCPharmer online tool (http://zincpharmer.csb.pitt.edu/pharmer.html). It was used to screen the ZINC purchasable database, which contains 35 million different compounds [65, 66]. The selected pharmacophore model (*Model 11*) (Fig. 5) was uploaded as a pharmacophore query; in addition, several filters were also used to ensure that only drug-like molecules are retrieved (350 ≤ molecular weight ≤ 500 *g*/mol) and (0 ≤ number of rotatable bonds ≤ 10) resulting in 6178 hits (Fig. 7).

Then, multiple filters were then used to keep only compounds with promising characteristics in the hit set. The filtration process was initially done using MOE software (Fig. 7). First, non-unique structures (compound duplicates) were eliminated, leaving 3467 compounds. Then, the Oprea’s lead-like filter was applied, removing non-lead-like compounds, leaving 2996 compounds [67]. Third, the mutagenic filter was applied to remove potential mutagenic compounds, leaving 2052 compounds [68]. Moreover, the compounds were also filtered using Veber’s rule for good oral bioavailability leaving 2034 molecules [69]. Finally, using MOE, the survived compounds were filtered according to their predicted solubility (logS) values, and molecules with a logS value less than − 5 were eliminated, leaving 904 compounds. Finally, PAINS-remover online tool (https://www.cbligand.org/PAINS/) was used to remove PAINS-containing hits, giving 890 compounds (Fig. 7) [70]. It is important to detect such compounds as they are likely to produce false positive results interfering with the results produced, especially when it comes to protein reactivity [70, 71]. Not only this, but those compounds represent poor candidates for drug development [70].

### ADME parameters and Pharmacokinetic properties assessment

The *SwissADME* online web tool (http://www.SwissADME.ch/index.php), offered by the *Swiss Institute of Bioinformatics (SIB)*, was used for the assessment of the pharmacokinetic characteristics of the survived 890 compounds [72–74]. This tool computes the different physicochemical descriptors and allows the prediction of ADME parameters, pharmacokinetic properties, drug-like nature, and medicinal chemistry friendliness [72]. This was important to limit the progression to the next step (molecular docking simulation) to the compounds with promising pharmacokinetic properties.

**Fig. 8:**
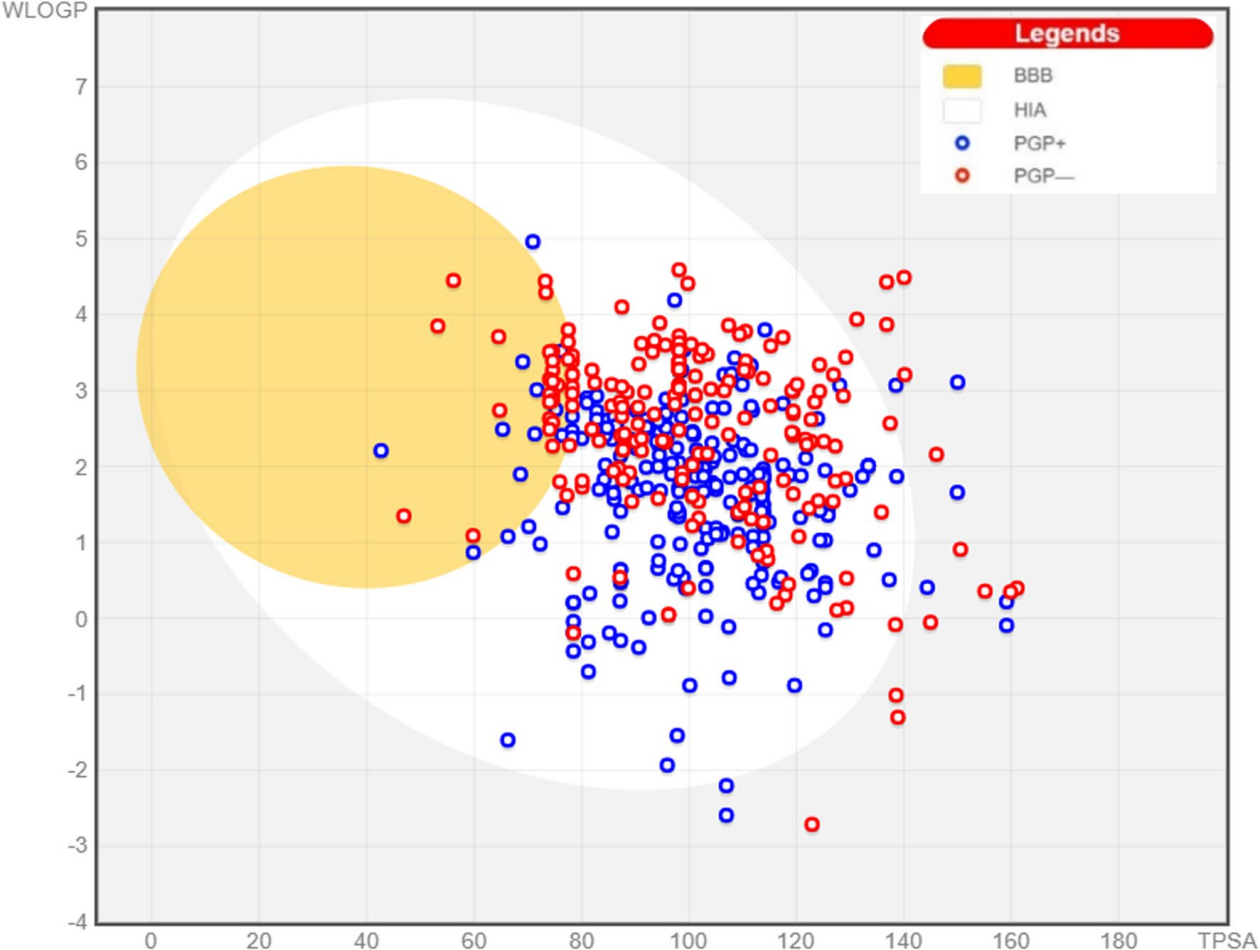
SwissADME Boiled-Egg plot for the compounds survived the filtration steps (only representative 400 molecules are shown for clarity)

Figure 8 shows the obtained *SwissADME* Boiled‒Egg plot, which estimates both the brain permeation and gastrointestinal (GIT) absorption of the tested molecules [72, 74]. This estimation is based on two physicochemical parameters, the wlogP and topological polar surface area (TPSA) [74]. The white region is the physicochemical space of molecules with highest probability of being absorbed by the GIT, and the yellow region (yolk) is the physicochemical space of molecules with highest probability to permeate to the brain. Compounds in blue are P-glycoprotein (P-gp) substrates, whereas compounds in red are not. Most of the tested compounds are likely to get absorbed through the GIT without passing the blood brain barrier (BBB); this in turn, prevents central side effects. Moreover, the plot shows that most of the tested compounds are not P-gp efflux protein substrates. P-gp substrates possess poor oral bioavailability and can induce or inhibit the P-gp function, reducing or increasing the bioavailability of other possible substrates, respectively [75].

Molecules were filtered accordingly to guarantee that only molecules with good pharmacokinetic properties were further processed. Out of the 890 compounds analysed, 50 molecules had low GIT absorption, 86 molecules displayed BBB permeability, and 386 molecules were P-gp substrates (some molecules have a combination of several factors). All those molecules were eliminated keeping 407 compounds. According to SwissADME predictions, the Abbott bioavailability scores of the survived compounds ranged from 0.55 to 0.56 indicating their good bioavailability and pharmacokinetic properties [76]. According to this score, those compounds are likely to have a bioavailability of greater than 10% in rats [76]. The synthetic feasibility scores also ranged from 2.2 up to 5.31 (on a scale of 1 (very easy) to 10 (very hard)) suggesting their ease of synthesis.

### Molecular docking simulations

To confirm the binding ability of the filtered hits in the binding sites of the target kinases, ROCK1 and ASK1, molecular docking simulations were performed in their kinase domains. Two ROCK1 and ASK1 protein structures were chosen from the training set based on their resolution. PDB ID: 4W7P with a resolution of 2.80Å (ROCK1), and PDB ID: 6VRE with a resolution of 2.29Å (ASK1). The docking protocol was first validated for each protein separately which indicated the suitability of the adopted docking protocol for the intended docking study. In ROCK1’s PDB ID: 4W7P self-docking, the docking pose has a docking score (S) of ‒15.15 kcal/mol and an RMSD value of 0.183Å from the co-crystalized pose achieving the essential interactions of the co-crystalised ligand. As for ASK1’s PDB ID: 6VRE self-docking, the docking pose has a docking score (S) of ‒13.38 kcal/mol and an RMSD value of 0.907Å from the co-crystalized pose performing all the key interactions of the co-crystalised ligand (See Additional file 1: S4 for further details).

The validated docking protocols were then used to perform the docking simulations for the survived 407 compounds in the binding sites of both target kinases. This step was done to simulate the molecules’ binding in the proteins’ binding sites, study their protein–ligand interactions, and predict their binding affinity. Moreover, to confirm the hits’ ability to satisfy the protein–ligand interactions proposed by the pharmacophoric features selected.

**Fig. 9:**
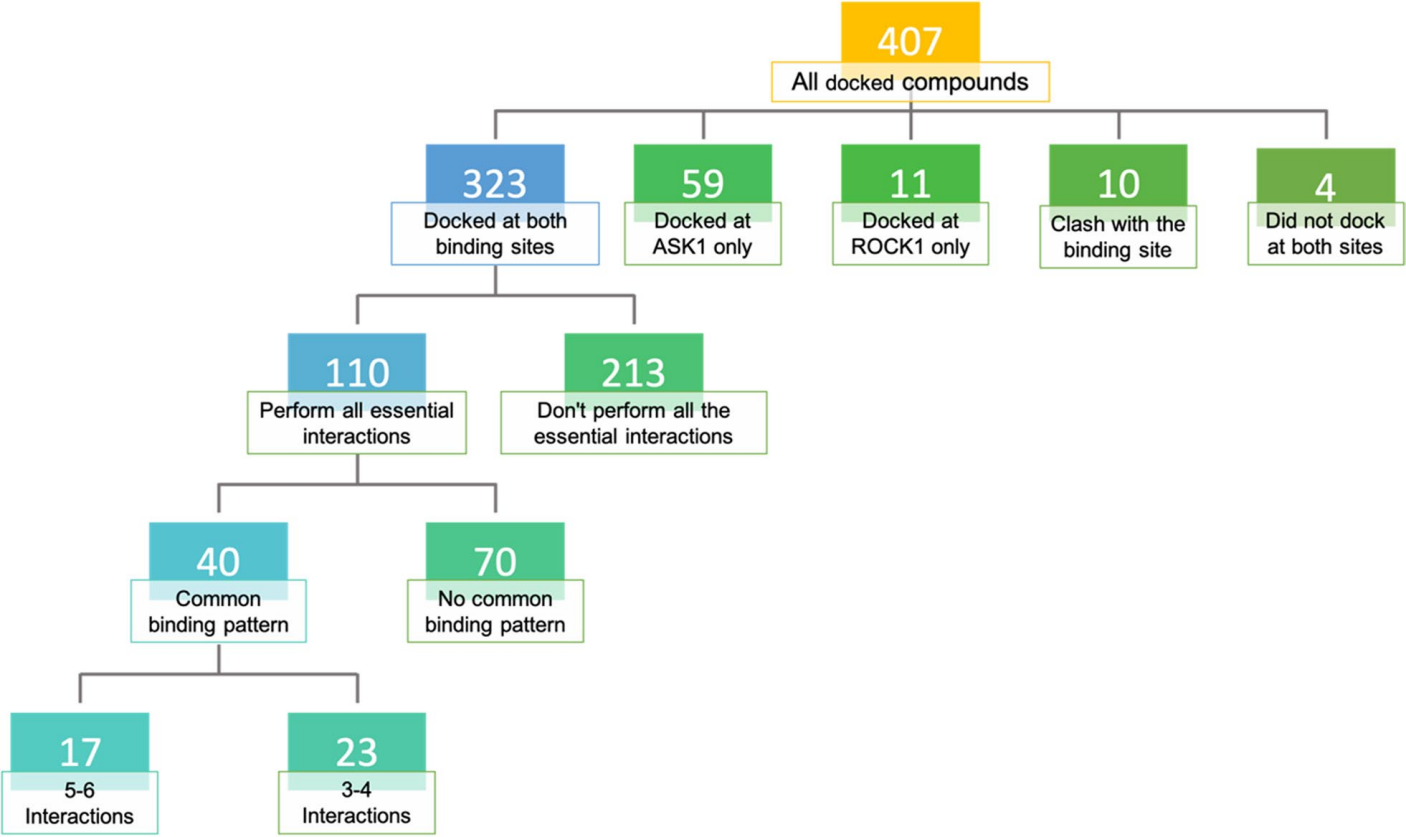
Classification of the 407 docked molecules based on their molecular docking results at the binding sites of the target kinases ROCK1 and ASK1

As can be seen in Fig. 9, out of the 407 molecules, 323 compounds had the potential to be docked in both protein structures (Dually docked compounds). On the other hand, 59 compounds were docked in ASK1 only, and 11 compounds were docked in ROCK1 only. Ten molecules were found to clash with either one or both binding sites and four compounds were not able to dock in neither of the binding sites.

Among the 323 dually docked molecules, the total number of interactions performed by each molecule varied, ranging from one to six interactions at either of the binding sites. Moreover, these dually docked molecules varied in their binding pattern. Upon deeper investigation of the binding pattern of the 323 dually docked compounds, 213 molecules were eliminated for not satisfying the essential interactions with either one or both proteins, which are at the hinge region with Tyr155 & Met156 (ROCK1) and Gln756 & Val757 (ASK1) and at the catalytic loop with Lys105 (ROCK1) and Lys709 (ASK1), leaving 110 promising molecules (Fig. 9).

The promising 110 molecules performing all the key interactions at the binding sites of the target kinases were then filtered leaving only the compounds that have a common binding pattern in both proteins leaving a total of 40 molecules (Fig. 9) (See Additional file 1: S5 [Model 11]. Those molecules were further categorized into two classes based on the number of interactions they performed at the binding sites of both proteins at − 0.1 kcal/mol energy cutoff. They were divided into two classes, 5–6 interactions and 3–4 interactions. The classes were divided based on the higher number of interactions observed at ROCK1 or ASK1 (Tables 8 and 9). It was apparent that some molecules were able to interact with additional amino acids at the hinge region (ROCK1: Glu154/ASK1: Glu755), DFG-motif (ROCK1: Asp216/ASK1: Asp822), and/or gatekeeper residues (ROCK1: Met153/ASK1: Met754). Thereby, those interactions were considered non-essential, aside from the essential interactions mentioned earlier.

**Table 8:** Docked compounds with 5–6 interactions at ROCK1 and/or ASK1 binding sites

		ROCK1		ASK1		Average**
Number of interactions	ZINC ID	No. of Interactions	Binding score*	No. of Interactions	Binding score*	Binding scores*
5–6 Interactions	ZINC04968098	5	‒13.13	3	‒13.81	‒13.47
	ZINC48234390	3	‒10.34	5	‒11.60	‒10.97
	ZINC12428844	3	‒10.67	5	‒11.09	‒10.88
	ZINC89779407	5	‒11.24	3	‒13.63	‒12.44
	ZINC20446787	5	‒12.20	5	‒13.29	‒12.74
	ZINC40129807	5	‒11.72	5	‒12.98	‒12.35
	ZINC32960731	5	‒12.72	3	‒12.27	‒12.50
	ZINC73988575	5	‒12.46	4	‒13.11	‒12.79
	ZINC72263787	3	‒12.76	5	‒15.29	‒14.03
	ZINC89638949	5	‒12.63	4	‒11.22	‒11.92
	ZINC05938451	5	‒13.41	4	‒11.95	‒12.68
	ZINC45947713	3	‒11.33	5	‒14.06	‒12.69
	ZINC66266481	3	‒10.72	5	‒11.43	‒11.07
	ZINC00637673	4	‒12.65	5	‒12.91	‒12.78
	ZINC77167529	4	‒10.86	5	‒11.71	‒11.28
	ZINC90394330	5	‒11.64	4	‒13.09	‒12.36
	ZINC60499106	5	‒11.16	5	‒12.36	‒11.76
**Co-crystalized ligands**		4	‒15.15	4	‒13.38	‒14.26

^*^In kcal/mol

^**^[(ROCK1 docking score + ASK1 docking score)/2]

**Table 9:** Docked compounds with 3–4 interactions at ROCK1 and/or ASK1 binding sites

Number of interactions		ROCK1		ASK1		Average**
	ZINC ID	No. of Interactions	Binding Score*	No. of Interactions	Binding Score*	Binding Scores*
3–4 Interactions	ZINC20978296	3	‒10.90	4	‒12.01	‒11.46
	ZINC72465685	3	‒12.20	4	‒12.54	‒12.37
	ZINC36693793	4	‒11.35	3	‒11.50	‒11.42
	ZINC78082037	4	‒12.14	3	‒11.64	‒11.89
	ZINC36693728	4	‒13.48	4	‒12.92	‒13.20
	ZINC04968107	4	‒10.74	3	‒12.83	‒11.79
	ZINC69582168	3	‒12.00	3	‒12.31	‒12.16
	ZINC69582176	3	‒10.60	3	‒11.43	‒11.01
	ZINC09503410	3	‒12.82	3	‒13.38	‒13.10
	ZINC29463219	3	‒12.86	3	‒13.34	‒13.10
	ZINC91041750	4	‒10.641	3	‒12.42	‒11.53
	ZINC56905939	4	‒12.91	3	‒12.39	‒12.65
	ZINC21466280	4	‒13.64	4	‒11.51	‒12.57
	ZINC77943297	3	‒10.53	4	‒12.98	‒11.76
	ZINC71715331	4	‒13.07	4	‒13.80	‒13.43
	ZINC04088663	3	‒13.99	3	‒13.84	‒13.91
	ZINC49028294	4	‒12.67	3	‒13.19	‒12.93
	ZINC72014041	4	‒13.35	4	‒13.30	‒13.33
	ZINC90422635	4	‒12.11	3	‒14.91	‒13.51
	ZINC64802850	3	‒12.51	3	‒13.12	‒12.81
	ZINC50059342	4	‒12.39	4	‒12.33	‒12.36
	ZINC91701857	3	‒11.18	3	‒13.83	‒12.50
	ZINC58364038	4	‒12.10	3	‒11.55	‒11.82
**Co-crystalized ligands**		4	‒15.15	4	‒13.38	‒14.26

^*^In kcal/mol

^**^[(ROCK1 docking score + ASK1 docking score)/2]

Furthermore, to be able to compare the overall performance of the different molecules at both binding sites; the average binding score was calculated for each molecule, allowing comparison with the self-docked co-crystalized ligands (Tables 8 and 9).

As can be seen in Tables 8 and 9, Seventeen molecules performed 5–6 interactions at either of the binding sites, whereas twenty-three molecules performed 3–4 interactions. Indicating the ability of some molecules to perform additional interactions at both binding sites increasing the selectivity and binding affinity towards the desired targets.

**Fig. 10:**
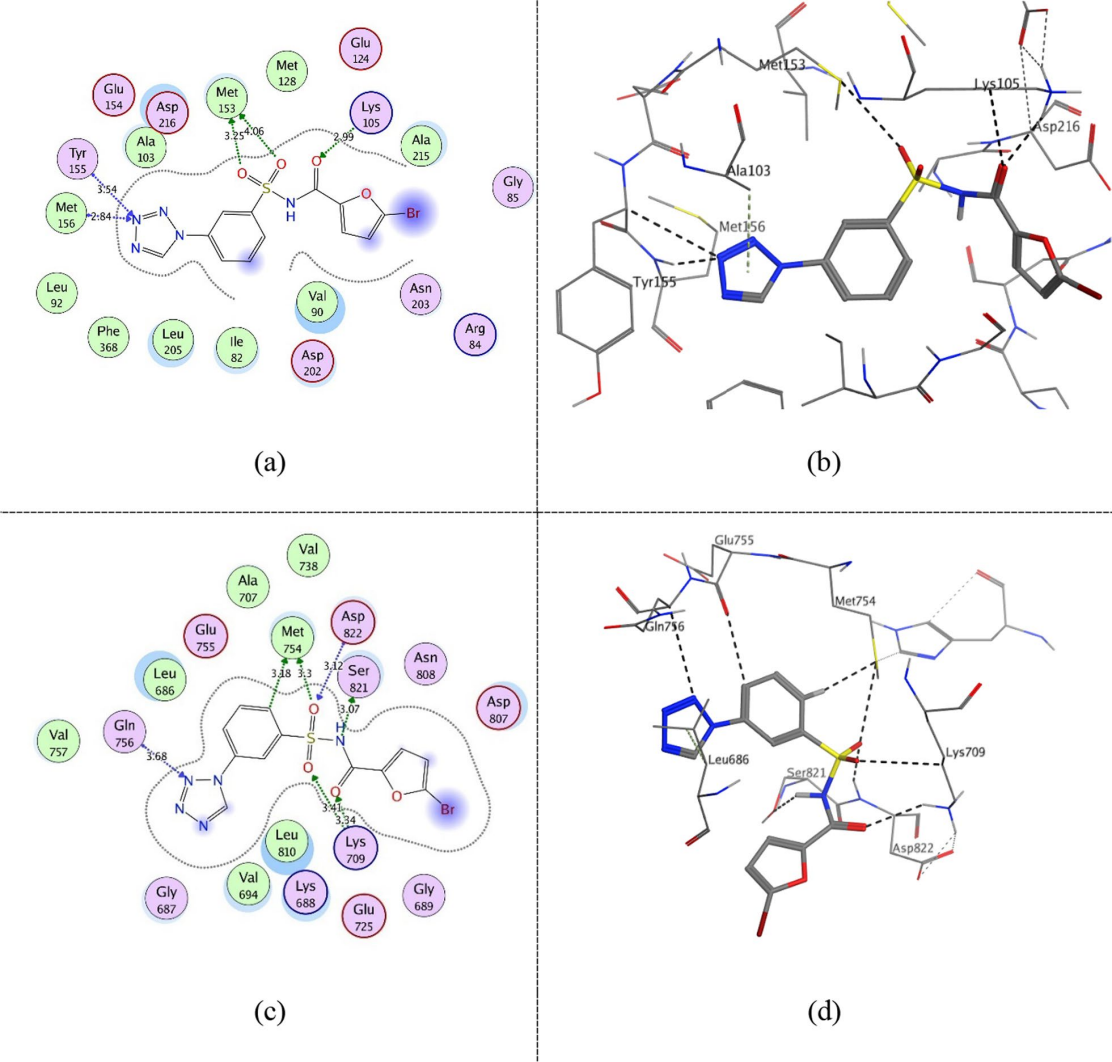
2D diagram (**a**) and 3D representation (**b**) of compound **ZINC60499106** interactions at − 0.3 kcal/mol energy cutoff in ROCK1 kinase domain (PDB ID: 4W7P) and 2D diagram (**c**) and 3D representation (**d**) of compound **ZINC60499106** interactions in ASK1 kinase domain (PDB ID: 6VRE)

**Fig. 11:**
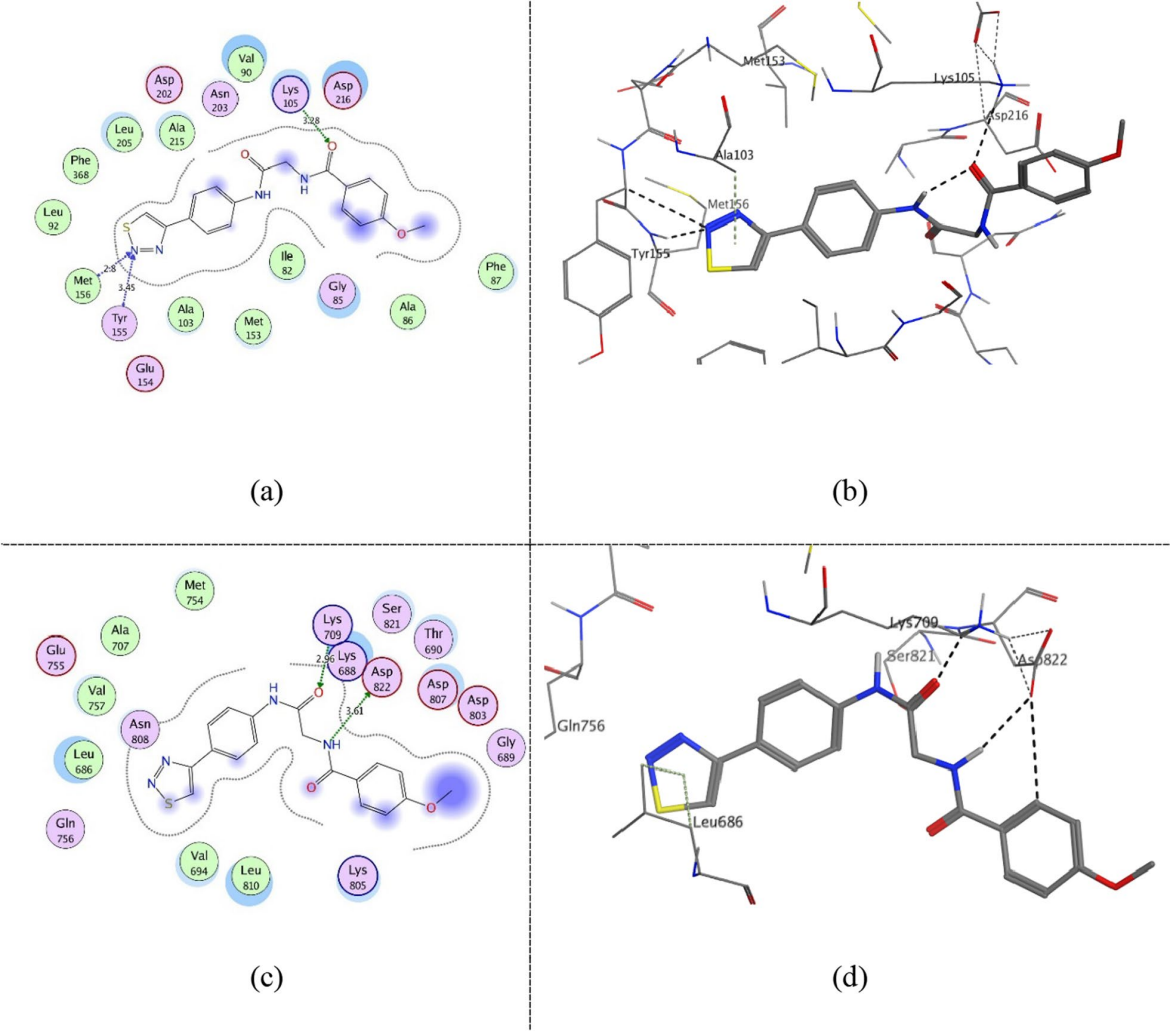
2D diagram (**a**) and 3D representation (**b**) of compound **ZINC69582168** interactions at − 0.3 kcal/mol energy cutoff in ROCK1 kinase domain (PDB ID: 4W7P) and 2D diagram (**c**) and 3D representation (**d**) of compound **ZINC69582168** interactions in ASK1 kinase domain (PDB ID: 6VRE)

**Fig. 12:**
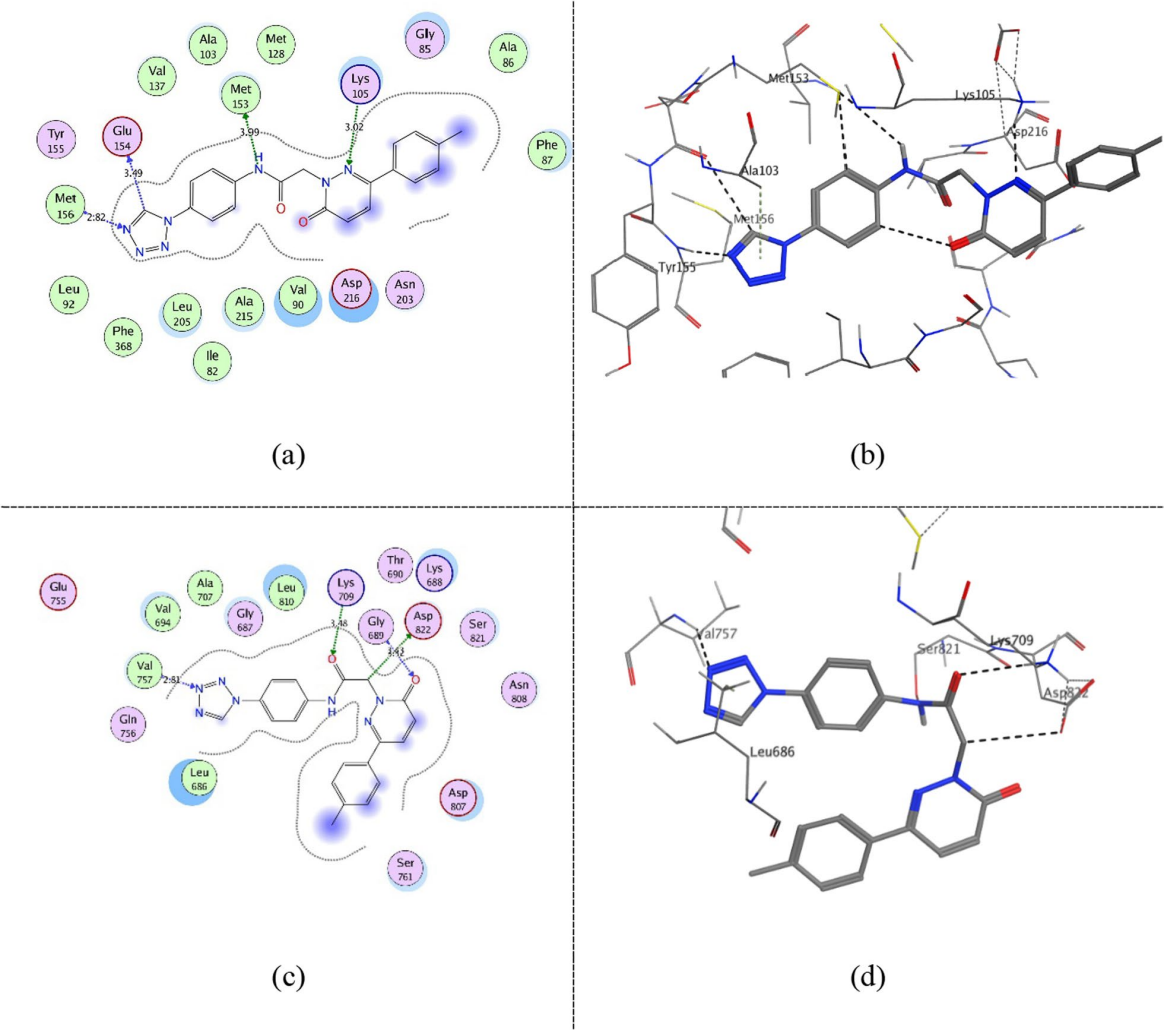
2D diagram (**a**) and 3D representation (**b**) of compound **ZINC04968098** interactions at − 0.3 kcal/mol energy cutoff in ROCK1 kinase domain (PDB ID: 4W7P) and 2D diagram (**c**) and 3D representation (**d**) of compound **ZINC04968098** interactions in ASK1 kinase domain (PDB ID: 6VRE)

Figure 10 shows compound **ZINC60499106** key interactions as type I inhibitor at − 0.3 kcal/mol energy cutoff at the kinase domain of both proteins as a representative for 5–6 interactions class. It interacts through hydrogen bonding with the catalytic (Lys105 (ROCK1)/Lys709 (ASK1)) via its hydrogen bond acceptor amide carbonyl group. Furthermore, in the hinge region, it performs hydrogen bonding with the key residues Tyr155 and Met156 (ROCK1)/Gln756 (ASK1) via its hydrogen bond acceptor tetrazole group. In addition, it interacts with the DFG-motif Asp822 (ASK1) via its hydrogen bond acceptor sulfoxide group. Furthermore, via its aromatic and heterocyclic rings, it is involved in several interactions at the binding pocket through its hydrophobicity and π electrons. Figure 11 shows compound **ZINC69582168** interactions at − 0.3 kcal/mol energy cutoff as a representative for 3–4 interactions class. Similarly, it interacts via its amide group with Lys105 (ROCK1)/ Lys709 (ASK1) while its thiadiazol group interacts with the previously mentioned residues of the hinge region. Figure 12 shows compound **ZINC04968098** interactions at − 0.3 kcal/mol energy cutoff as another representative for 5–6 interactions class. Through its amide and pyridazine ring, it interacts via hydrogen bonding with the catalytic (Lys105 (ROCK1)/Lys709 (ASK1)) and via its tetrazole group with the hinge region residues. Moreover, an additional hydrogen bond interaction is observed between the amide group nitrogen and Met153 gatekeeper residue in ROCK1 binding site.

### Molecular dynamics simulations

**Fig. 13:**
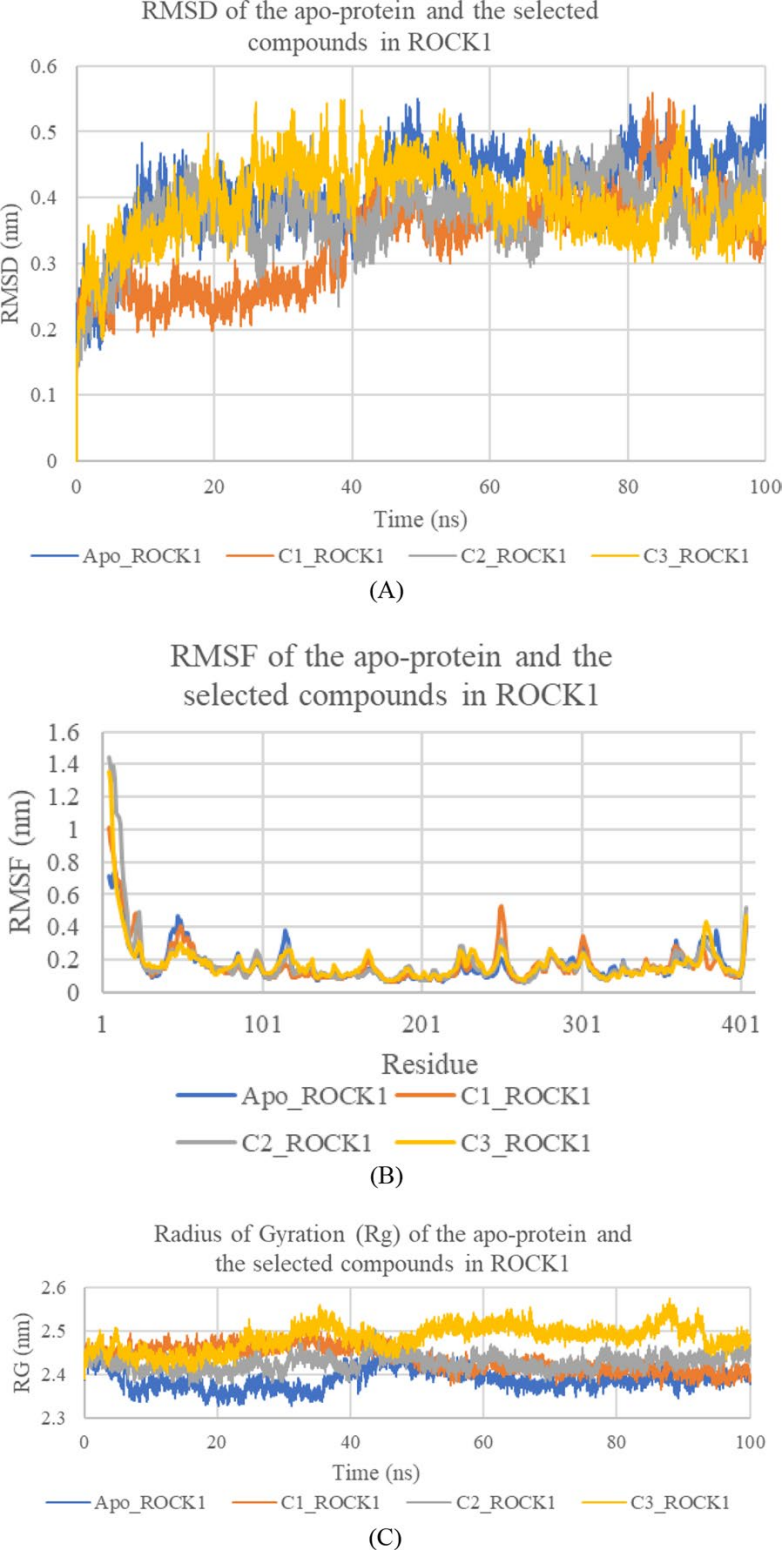
RMSD (**a**) RMSF (**b**) and Radius of Gyration (RG) (**c**) of the apo-protein and the selected three compounds in ROCK1. Apo_ROCK1: Apo-protein, C1_ROCK1: **ZINC60499106/ROCK1** complex, C2_ROCK1: **ZINC69582168/ROCK1** complex, and C3_ROCK1: **ZINC04968098/ ROCK1** complex

**Fig. 14:**
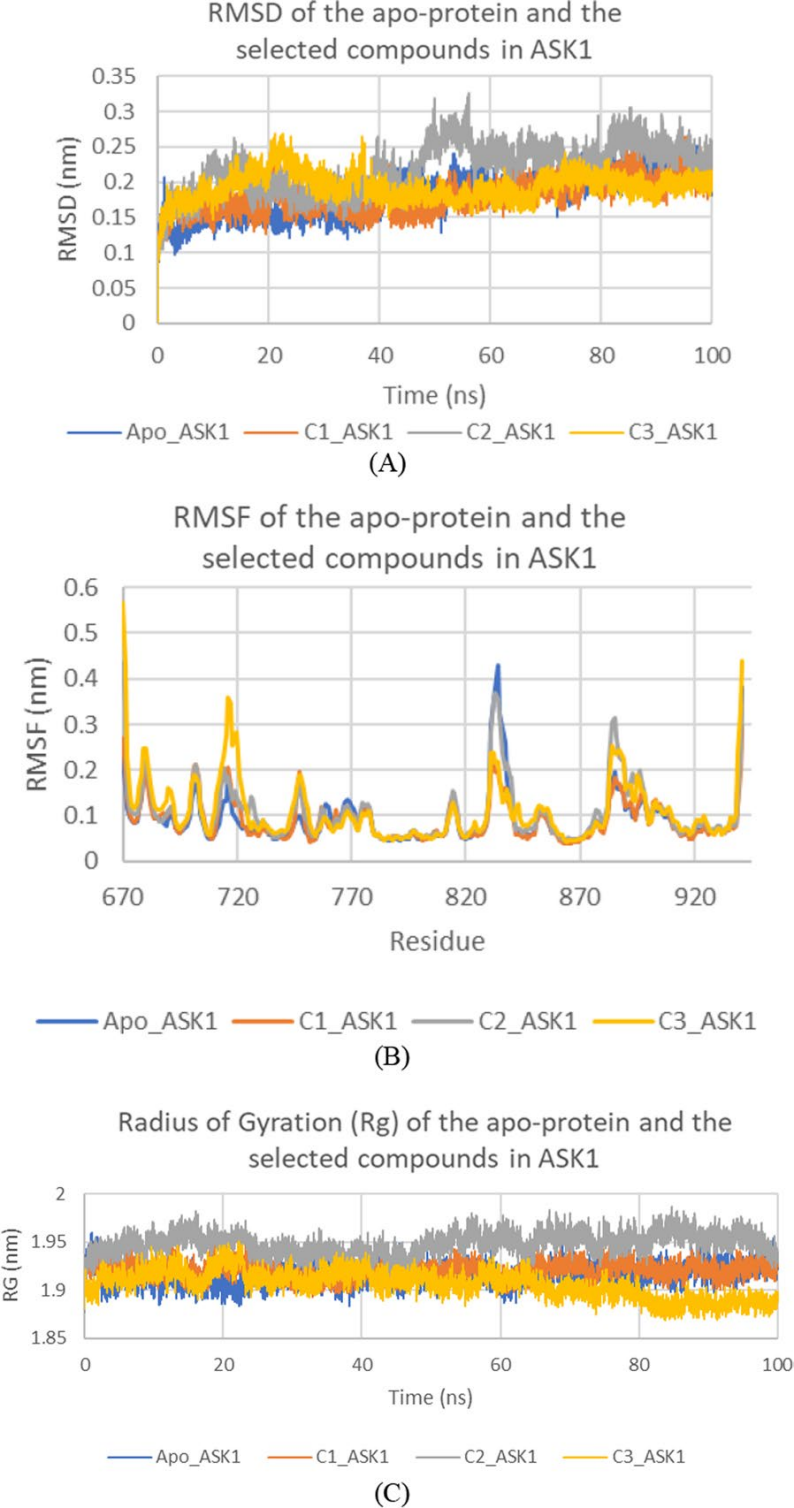
RMSD (**a**) RMSF (**b**) and Radius of Gyration (RG) (**c**) of the apo-protein and the selected three compounds in ASK1 (**b**) and Radius of Gyration (RG) of the apo-protein and the three compounds. Apo_ASK1: Apo-protein, C1_ASK1: **ZINC60499106/ASK1** complex, C2_ASK1: **ZINC69582168/ASK1** complex, and C3_ASK1: **ZINC04968098/ASK1** complex

The dynamic behaviour and stability of the three representative compounds ZINC60499106, ZINC69582168, and ZINC04968098 in the target kinases were investigated via running molecular dynamics simulations of each compound in both ROCK1 and ASK1 for 100 ns starting from the docking geometries. In ROCK1 simulations, inspection of the RMSD of the apo-protein and the bound complexes of the three compounds along the 100 ns trajectories showed that the trajectories of the apo-protein, ZINC69582168, and ZINC04968098 in ROCK1 complexes, stabilized after about 20 ns of the simulation time, whereas ZINC60499106-ROCK1 complex reached equilibrium after about 40 ns. As for ASK1 simulations, RMSD results showed that the apo-protein, ZINC60499106-ASK1, ZINC04968098-ASK1 complexes stabilized after about 20 ns, whereas the ZINC69582168-ASK1 system showed slight fluctuation at about 50 ns, then remained stable till the end of the simulation time. The RMSD results in both target kinases show the stability of the systems as reflected by the small RMSD values. On calculating Root Mean Square Fluctuation (RMSF) values, it was found that apart from the peripheral terminal residues, the complexes show very low fluctuations which were less than or equal to about 0.5 nm during the simulation time in both ROCK1 and ASK1 target kinases. According to the Radius of Gyration (Rg) calculation, the compactness of both the ROCK1 and ASK1 kinases were found to remain relatively constant throughout the simulations, reflecting their stable conformations (Figs. 13 and 14). Altogether, the results suggest the stability of the systems. Interestingly, on visualizing the simulation trajectories, it was found that further interactions with Lys688 in ASK1 were established beside the interactions shown in the docking study.

### Clustering

Virtual screening usually yields hits with high structural similarity; therefore, the compounds showed promising docking results were clustered based on their structural similarity to identify the privileged scaffolds for dual ROCK1/ASK1 inhibition. The clustering process was done using *DataWarrior 5.5.0* [77]. This program works by using different descriptors to form and visualize the chemical space of the uploaded database [77]. This allows the exploration of the dataset chemical space further [77]. Clusters were then formed with the highest similarity set to fall below 60%. The 110 compounds performing the essential interactions were clustered forming a set of 33 clusters, the largest clusters formed are shown in Table 10. It is apparent from the obtained clusters that the central amino groups are essential for inhibition at both binding sites. The presence of an aromatic ring at either side of the amine group is likely to be essential as well for performing aromatic hydrophobic interactions with the corresponding amino acids within the binding site. Moreover, the presence of a hydrogen bond acceptor (electron-donating) group such as the tetrazole ring, thiophene ring, sulfoxide, and carboxylate groups is essential for hydrogen bond interaction with the catalytic lysine amino acid .

**Table 10:** The representative compounds of the largest 6 clusters for the 110 compounds achieved the key interactions in ROCK1 and ASK1

Cluster Number	Cluster representative	Total number of compounds within the cluster
2		7
3		10
6		13
8		7
11		7
18		9

Similarly, the forty compounds that had a common binding pattern at both binding sites were clustered to determine if there are any commonly observed scaffolds required for this specific binding pattern. This is important for further development of dual ROCK1/ASK1 inhibitors and a total of 17 clusters were produced (Table 11). It is worth noting that the clusters composed of the largest set of compounds most likely represent promising ROCK1/ASK1 dual inhibitors. The compounds possessing a common binding pattern shared the central amine groups as well. One of the amine groups can be as well replaced with a sulfoxide group giving the hydrogen bond feature near the centre of the molecule as depicted by the pharmacophore model. The presence of the aromatic rings as well is portrayed to be essential for the aromatic π and hydrophobic interactions between the corresponding inhibitors and the binding site amino acids. The thiophene and tetrazole rings as well reoccur in the molecules with a common binding pattern allowing the hydrogen bond interaction with the catalytic lysine residue.

**Table 11:** The representative compounds of the largest 3 clusters for the 40 compounds with the common binding pattern in ROCK1 and ASK1

Cluster Number	Cluster representative	Total number of compounds within the cluster
1		4
5		7
6		8

## Conclusion

Due to the lack of approved treatments, NASH poses burden on both patients and healthcare systems. However, protein kinases such as ROCK1 and ASK1 are involved in the progression of the disease and are promising targets for therapy creating an area of possible dug discovery. Furthermore, their sequence, topological and structural similarity indicates their potential for dual inhibition.

Receptor-based pharmacophore modeling was initially used to generate a pharmacophore model representing the common structural features observed in both ROCK1 and ASK1 type 1 (ATP-competitive) inhibitors. Thirteen pharmacophore models were generated and were evaluated using a test set of thirty-nine ROCK1 and ASK1 inhibitors along with 1198 decoys. Various assessment metrics were used to compare the models’ ability to discriminate between actives and decoys reflecting their validity in virtual screening. Pharmacophore model 11 was the best model achieving an MCC value of 0.71. This model consisted of three acceptor and two aromatic features representing the main features for binding of both ROCK1 and ASK1 inhibitors.

The selected model was then used to screen the ZINC purchasable database using the ZINCPharmer webtool resulting in 6178 hits. They underwent several medicinal chemistry filtration steps giving 890 molecules. To confirm that those hits possess promising pharmacokinetics and binding characteristics in ROCK1 and ASK1 kinase domains, they were first filtered according to their pharmacokinetic properties using the *SwissADME* webtool resulting in 407 promising compounds. The survived molecules were then subjected to molecular docking into ROCK1 and ASK1 kinase domains. Interestingly, 110 compounds were found to perform all the essential interactions required at both kinase binding sites. Moreover, 40 compounds of which were found to possess a common binding pattern at both binding sites. Clustering of the promising compounds was carried out to determine the privileged scaffolds for ROCK1/ASK1 dual inhibition to aid further in the discovery and development. Upon clustering, it was apparent that the central amino groups are essential for inhibition at both binding sites. The presence of an aromatic ring at either side of the amine group is likely to be essential as well for performing aromatic hydrophobic interactions with the corresponding amino acids within the binding site. Moreover, the presence of a hydrogen bond acceptor (electron-donating) group such as the tetrazole ring, thiophene ring, sulfoxide, and carboxylate groups is essential for hydrogen bond interaction with the catalytic lysine group.

The outlook of the current work is that the most promising molecules are to be tested in vitro on ROCK1 and ASK1 enzymes and on hepatic cells and in vivo using NASH animal models.

## Methods

All molecular modeling studies were carried out using the Molecular Operating Environment Software (MOE 2022.02), unless otherwise stated.

### Selection of the X-ray crystallographic structures for ROCK1 and ASK1 and training set generation

The X-ray crystal structures of ROCK1 and ASK1 were first downloaded from the Protein Data Bank (PDB) (https://www.rcsb.org/). The PDB search resulted in twenty-three ROCK1 structures and nineteen ASK1 structures. Protein structures bound to non-druglike ligands or ligands with a unique binding pattern were eliminated. The protein structures were then clustered according to their inhibitors’ binding pattern (Redundant structures containing ligands with highly similar structure to those in pre-clustered PDB structures were eliminated). The cluster with the most common binding pattern in case of each protein was selected. This gave a set of three ROCK1 and four ASK1 proteins (Tables 2 and 3) (See Additional file 1: S1 for further details).

For each crystal structure selected, the best-quality protein chain was chosen to represent the corresponding protein crystal structure (Table 12). Furthermore, ligands and water molecules that are unnecessary for ligand binding were removed. The protein structures were then prepared using the *QuickPrep* tool in MOE.

**Table 12:** Protein structures' preparation procedure

PDB ID	Preparation
7JOU	Chain A was kept
4YVC	Chain B was removed, keeping chain A
4W7P	Chains A, B, and C were removed, keeping chain D
6VRE	Chain B was removed, keeping chain A
5V24	Chain A was removed, keeping chain B
5VIL	Chains A, C, and D were removed, keeping chain B
5UOX	Chain B was removed, keeping chain A

### Test set generation

Two separate test sets were compiled one for each protein. Regarding ROCK1, a pre-compiled test set was available on DEKOIS 2.0 (http://www.pharmchem.uni-tuebingen.de/dekois/) [57]. It consists of forty active compounds and 1200 decoys. The test set’s active inhibitors were then filtered based on their activity level and molecular weight to comply with drug-like properties. Only molecules with molecular weight within the 350–500 Da range and IC_50_ values less than 10 μM were kept, resulting in a set of 20 active compounds (Table 4). To mimic the natural chemical space ratio between active and inactive compounds (> 1:30) [62], six hundred drug-like decoys were randomly selected for the test set from the original DEKOIS 2.0 test set [62].

As for ASK1, there is not any pre-compiled test set available in the searched databases, thus, a set of active compounds was self-collected. This set was retrieved from the PDB and several additional research papers [46, 47, 56, 58–60]. This set was then filtered using the same parameters for molecular weight (350–500 Da) and activity (IC_50_ < 10 μM), resulting in a set of 20 active ASK1 inhibitors (Table 5). The decoys were then generated using DUD-E (http://dude.docking.org/generate), using the self-collected active molecules as the nucleus, returning 1250 decoys which were filtered randomly to retrieve a set of 600 drug-like decoys [61].

Energy minimization was carried out for both test sets using MOE at an RMS gradient of 0.1 kcal mol^−1^Å^−2^ with *Amber10* force field and with automatically calculated partial charges. The compounds’ ionization states were computationally predicted at the physiological pH using the database *wash* function in MOE. Following this, conformational search was carried out for all the test set compounds using the *LowModeMD* method, which is the default option in MOE conformational search due to its overall best performance compared to other methods. for the ROCK1 test set, this resulted in 1979 conformers of the active compounds (19/20 compounds) and 44,856 conformers of the decoys (598/600 compounds), and for the ASK1 test set, 2591 conformers of the active compounds (20/20 compounds) and 48,813 conformers of the decoys (600/600 compounds).

### Pharmacophore models generation

The prepared ROCK1 and ASK1 protein crystal structures and their co-crystalized inhibitors were aligned and superimposed using the MOE *Align* and *Superimpose* protocols, respectively. Following this, the aligned ligands were used to manually generate receptor-based 3D pharmacophore models using *Pharmacophore query editor* in MOE. Those models were generated based on the common interactions observed between the inhibitors and the aligned proteins. The common interactions observed included H-bond interactions with the hinge region residues (ROCK1 Tyr155 and Met156; ASK1 Gln756 and Val757) and the key catalytic lysine residue (ROCK1 Lys105; ASK1 Lys709). Moreover, there were also common arene interactions with the gate area residues (ROCK1 Val90; ASK1 Val694).

Furthermore, excluded volumes were also used to define the steric extent of the proteins’ binding sites, which were generated automatically using the MOE *pharmacophore query editor* using the ROCK1 (PDB ID: 4W7P) binding site. For further fine-tuning of the pharmacophore models’ quality, additional tailored excluded volumes were added manually. 4W7P’s pocket was chosen due to its large size reflected by binding to the largest inhibitor of the training set.

### Pharmacophore model selection and validation

The manually generated pharmacophore models were then used for screening the prepared test sets of both ROCK1 and ASK1. This was done to assess the ability of the generated pharmacophore models to distinguish between active and inactive compounds. MOE pharmacophore search algorithm first prefilters the generated conformers based on two main characteristics: feature type, and distance similarity to the mapped pharmacophore model. Following this, further expensive alignment between the different conformers and the query feature points is done to minimize their deviation from each other.

The screening output of the test sets [True positive (TP), true negative (TN), false positive (FP), and false negative (FN)] was used to calculate several assessment metrics to select the model with the best performance in terms of discriminating between actives and inactives. These metrics include sensitivity (Se), specificity (Sp), yield of actives (Ya), enrichment (E), accuracy (acc), discrimination ratio (DR), F1 score (F1), and Mathew’s correlation coefficient (MCC) (For further details see Additional file 1: S2).

### Virtual screening and hit filtration

Virtual screening was conducted using the best-performing pharmacophore model. The screening was done using the ZINC purchasable database which contains over 35 million compounds [52]. The online ZINCPharmer web tool was used to perform the screening process [65]. This tool is a pharmacophore search web tool that can be used for screening the ZINC purchasable database (http://zincpharmer.csb.pitt.edu/). To limit the number of hits produced and to ensure that they have drug-like properties, two additional filters were used. The first filter set the hits’ molecular weight to be within 350 and 500 Da (350 ≤ molecular weight ≤ 500 Da), and the second filter limits the number of rotatable bonds in the obtained hits from 0 to 10 [62].

Following the virtual screening process, the produced hits were filtered further to ensure that only lead-like molecules were selected for further steps. The duplicate compounds were first removed, then five different filters were applied (Table 13). Four MOE filters were applied in sequence, first the Oprea’s lead-like filter removing compounds with non-lead-like properties [67]. Secondly, potential mutagenic molecules were identified and removed through the screening for certain molecular characteristics, according to Kazius et al. [68]. Moreover, topological polar surface area (TPSA) was also calculated to check the molecules’ compliance with Veber’s rule (TPSA < 140Å^2^), and non-compliant molecules were removed [69]. LogS was also calculated, and molecules which do not fall within the lead-like value range (≥ − 5) were eliminated [78]. Finally, the Pan-Assay Interference compounds (PAINS), which are known to be promiscuous molecules with multiple behaviours that interfere with assay readouts, were removed using the online PAINS removal tool (https://www.cbligand.org/PAINS/) [70].

**Table 13:** The different criteria for hit filtration

Criterion	Cutoff value
**Oprea’s lead-like filter**	
Molecular weight	< 450
logP	≤ 4.5
Number of hydrogen bond acceptors	≤ 8
Number of hydrogen bond donors	≤ 5
**Mutagenic molecules filter**	= 0
**Veber’s rule**	
Topological polar surface area	< 140 Å^2^
**logS**	≥ − 5
**PAINS Filter**	No

### ADME parameters and pharmacokinetic properties assessment

The *SwissADME* online tool, created by the *Swiss Institute of Bioinformatics* (SIB), was used (http://www.SwissADME.ch/) to assess the molecules separately [72–74]. This tool calculates individual molecular physicochemical descriptors and estimates ADME parameters, pharmacokinetic properties, drug-like nature, and medicinal chemistry amiability [72]. To process the molecules, they were first directly placed in the form of SMILES in the input box, along with their corresponding ZINC IDs. Following this, the molecules were submitted to the online server for processing. The results were then obtained on the submission page of *SwissADME*. Molecules with promising pharmacokinetic properties according to their GIT absorption, BBB permeation, and P-gp susceptibility are selected for further progression (Table 14).

**Table 14:** The criteria used to filter molecules according to their pharmacokinetic properties

Criterion	Category
GIT absorption	High
BBB permeation	No
P-gp substrate	No

### Molecular docking simulations

ROCK1 and ASK1 X-ray crystal structures with the best resolution in the training set were chosen for the molecular docking studies. PDB ID: 4W7P (Resolution = 2.8 Å) and PDB ID: 6VRE (Resolution = 2.29 Å) were chosen for ROCK1 and ASK1 molecular docking studies, respectively.

The docking protocol was first validated for both proteins. The validation was carried out by self-docking of the co-crystallized ligand in each protein structure in their respective binding sites. Amber10 force field, *Triangle Matcher* placement method, *Rigid Receptor* refinement method, and *London dG* scoring function were used to generate different possible docking poses. In ROCK1, PDB ID: 4W7P, the docking pose produced by the adopted docking protocol had a score (S) of ‒15.15 kcal/mol and an RMSD value of 0.183Å, achieving the essential interactions of the co-crystalized ligand. On the other hand, the docking pose produced by the self-docking of the co-crystalized ligand in ASK1, PDB ID: 6VRE produced a pose with a score (S) of ‒13.38 kcal/mol and an RMSD value of 0.907Å performing all the key ligand interactions of the co-crystalized ligand (See Additional file 1: S4 for further details). These results indicate the suitability of the adopted docking protocol for the intended docking study.

The molecules which survived the previous filtration steps were docked into each protein using a two-feature pharmacophore containing two acceptor features, one at the hinge region and another at the catalytic lysine residues to speed up the docking process and restrict the docking poses to those achieving the key interactions.

The molecules that were docked into both binding sites and performed all the key interactions were then categorized into different classes based on the number of interactions they performed within the binding sites at − 0.1 kcal/mol energy cutoff. The molecules bind at both sites with the same binding pattern were isolated as well.

### Molecular dynamics simulations

To further examine the binding mode of the three representative compounds ZINC60499106, ZINC69582168, and ZINC04968098, we performed molecular dynamics simulations for them in both ROCK1 and ASK1 as well as for the apo-proteins. All simulations were performed using the GROMACS 2021.3 (Groningen Machine for Chemical Simulations) package [79]. Protein topology was generated using Amber99SB force field [80]. The three ligands were parametrized with Amber GAFF force field [81, 82]. To generate ligands’ topologies, ACPYPE (AnteChamber Python Parser Interface) [83] was used. Solvation of all systems were performed in a triclinic box of 1 nm size with the Tip3P water model, and they were neutralized with Na^+^ and Cl^−^ ions. Steepest descent algorithm was used to energy minimize the systems, until it converged with F_max_ not exceeding 1000 kJ mol^−1^ nm^−1^. The systems were then equilibrated under NVT and NPT ensembles for 100 ps each, while restraining the protein atomic positions. The velocity rescale (V-rescale) thermostat [84] with a time constant of 0.1 ps, was used to keep the temperature at 300 K. The pressure was maintained isotropically in the simulations, at 1 bar. using the Berendsen pressure coupling method during NPT equilibration. During the full production runs, the Parrinello − Rahman barostat [85] was used with a time constant of 2 ps. The Linear Constraint Solver (LINCS) algorithm [86] was used for all bonds constraining, allowing for an integration time step of 2 fs. Long-range electrostatics were described using the Particle Mesh Ewald summation (PME) method [87]. Both, the long-range electrostatic cut-off and the short-range van der Waals cut-off were set at 1 nm. Production runs for the equilibrated systems were done for 100 ns each using the leap-frog algorithm with a timestep of 2 fs. The analysis of the resulting trajectories was performed using in- GROMACS tools, and they were visualized with Chimera 1.16 [88].

### Clustering

The molecules achieved the essential interactions and ones with the common binding pattern were both clustered separately to determine the privileged scaffolds for ROCK1/ASK1 dual inhibition to aid further in the discovery and development. The compounds were clustered using the *clustering algorithm* in DataWarrior 5.5.0. [77]. This algorithm considers the structural similarity between molecules and works by first calculating the similarity matrix for the whole set of compounds. Then, the most similar compounds are joined together to form the first cluster. The similarity values of compounds forming the first cluster are then removed, and the similarities are calculated again to form the second cluster. The process repeats itself and joins the most similar compounds. It stops when one of two conditions or stop criterion is reached. It either stops when it reaches a predefined number of clusters or when the similarity falls below a predefined limit. In this study, the second stop criterion was used, and the similarity limit value was set to 60%. After the clusters’ formation, a representative hit compound from each cluster was randomly selected.

### Supplementary Information

**Additional file 1.** **S1.** Selection of the X-ray crystallographic structures for ROCK1 and ASK1 and training set generation. **S2.** Assessment metrics used to assess the performance of the constructed pharmacophore models. **S3.** The quantitative and qualitative description of the generated Pharmacophore models. **S4.** Self-docking validation of the used molecular docking protocol. **S5.** Common binding pattern molecules mapped onto the chosen pharmacophore model.

## Data Availability

Supplementary material word file is available in the online version of this article.

## Abbreviations

Acc: Acceptor feature
ACG: Protein kinase A, G, and C family
Aro: Aromatic feature
ASK1: Apoptosis Signal–Regulating Kinase 1
BBB: Blood brain barrier
BLAST: Basic Local Alignment Search Tool
DFG: Asp-Phe-Gly motif
DUD: Directory of useful decoys
GIT: Gastrointestinal tract
MAPK: Mitogen-activated protein kinase
MCC: Mathew’s correlation coefficient
MOE: Molecular Operating Environment software
NAFLD: Non-alcoholic fatty liver disease
NASH: Non-alcoholic steatohepatitis
PAINS: Pan-assay interference compounds
PDB: Protein Data Bank
P-gP: P-glycoprotein
PK: Protein kinase
RMSD: Root man square deviation
ROCK1: Rho-Associated Protein Kinase 1
TPSA: Topological polar surface area

## References

[1] Younossi ZM, Koenig AB, Abdelatif D, Fazel Y, Henry L, Wymer M (2016). Global epidemiology of nonalcoholic fatty liver disease—meta-analytic assessment of prevalence, incidence, and outcomes. Hepatology.

[2] Huang DQ, El-Serag HB, Loomba R (2021). Global epidemiology of NAFLD-related HCC: trends, predictions, risk factors and prevention. Nat Rev Gastroenterol Hepatol.

[3] Tomah S, Mohamed Eid E, Abouelmagd MM, Hassan AH, Eldib AH, Hamdy O (2019). 214-LB: vibration-controlled transient elastography reveals alarming prevalence of nonalcoholic fatty liver disease and fibrosis among young adults in Egypt. Diabetes.

[4] Abd El-Kader SM, El-Den Ashmawy EMS (2015). Non-alcoholic fatty liver disease: the diagnosis and management. World J Hepatol.

[5] MSD Australia. Nonalcoholic fatty liver disease (NAFLD)—hepatic and biliary disorders—MSD manual professional edition. MSD manual professional edition. 2021. https://www.msdmanuals.com/professional/hepatic-and-biliary-disorders/approach-to-the-patient-with-liver-disease/nonalcoholic-fatty-liver-disease-nafld. Accessed 27 Jul 2022.

[6] Puri P, Sanyal AJ (2012). Nonalcoholic fatty liver disease: definitions, risk factors, and workup. Clin Liver Dis.

[7] Adams LA, Feldstein AE (2010). Nonalcoholic steatohepatitis: risk factors and diagnosis. Expert Rev Gastroenterol Hepatol.

[8] Tiniakos DG, Vos MB, Brunt EM (2010). Nonalcoholic fatty liver disease: pathology and pathogenesis. Annu Rev Pathol.

[9] Loomba R, Adams LA (2019). The 20% rule of NASH progression: the natural history of advanced fibrosis and cirrhosis caused by NASH. Hepatology.

[10] Kopec KL, Burns D (2011). Nonalcoholic fatty liver disease: a review of the spectrum of disease, diagnosis, and therapy. Nutr Clin Pract.

[11] Promrat K, Kleiner DE, Niemeier HM, Jackvony E, Kearns M, Wands JR (2010). Randomized controlled trial testing the effects of weight loss on nonalcoholic steatohepatitis. Hepatology.

[12] Mummadi RR, Kasturi KS, Chennareddygari S, Sood GK (2008). Effect of bariatric surgery on nonalcoholic fatty liver disease: systematic review and meta-analysis. Clin Gastroenterol Hepatol.

[13] Esler WP, Bence KK (2019). Metabolic targets in nonalcoholic fatty liver disease. Cell Mol Gastroenterol Hepatol.

[14] Alshehade S, Alshawsh MA, Murugaiyah V, Asif M, Alshehade O, Almoustafa H (2022). The role of protein kinases as key drivers of metabolic dysfunction-associated fatty liver disease progression: new insights and future directions. Life Sci.

[15] Ibrahim SH, Hirsova P, Malhi H, Gores GJ (2020). Nonalcoholic steatohepatitis promoting kinases. Semin Liver Dis.

[16] Knighton DR, Zheng J, ten Eyck LF, Ashford VA, Xuong NH, Taylor SS (1991). Crystal structure of the catalytic subunit of cyclic adenosine monophosphate-dependent protein kinase. Science.

[17] Roskoski R (2019). Cyclin-dependent protein serine/threonine kinase inhibitors as anticancer drugs. Pharmacol Res.

[18] Taylor SS, Kornev AP (2011). Protein kinases: evolution of dynamic regulatory proteins. Trends Biochem Sci.

[19] Modi V, Dunbrack RL (2019). Defining a new nomenclature for the structures of active and inactive kinases. Proc Natl Acad Sci U S A.

[20] Adams JA (2001). Kinetic and catalytic mechanisms of protein kinases. Chem Rev.

[21] Roskoski R (2016). Classification of small molecule protein kinase inhibitors based upon the structures of their drug-enzyme complexes. Pharmacol Res.

[22] Dar AC, Shokat KM (2011). The evolution of protein kinase inhibitors from antagonists to agonists of cellular signaling. Annu Rev Biochem.

[23] Zuccotto F, Ardini E, Casale E, Angiolini M (2010). Through the “gatekeeper door”: Exploiting the active kinase conformation. J Med Chem.

[24] Nagar B, Bornmann WG, Pellicena P, Schindler T, Veach DR, Miller WT, et al. Crystal structures of the kinase domain of c-Abl in complex with the small molecule inhibitors PD173955 and imatinib (STI-571)—PubMed. 2002. https://pubmed.ncbi.nlm.nih.gov/12154025/. Accessed 3 Oct 2022. 12154025

[25] Backes AC, Zech B, Felber B, Klebl B, Müller G (2008). Small-molecule inhibitors binding to protein kinases. Part I: exceptions from the traditional pharmacophore approach of type I inhibition. Expert Opin Drug Discov.

[26] Liao JJ, lou.  (2007). Molecular recognition of protein kinase binding pockets for design of potent and selective kinase inhibitors. J Med Chem.

[27] Pearce LR, Komander D, Alessi DR (2010). The nuts and bolts of AGC protein kinases. Nat Rev Mol Cell Biol.

[28] Huang Z, Wu LM, Zhang JL, Sabri A, Wang SJ, Qin GJ (2019). Dual specificity phosphatase 12 regulates hepatic lipid metabolism through inhibition of the lipogenesis and apoptosis signal-regulating kinase 1 pathways. Hepatology.

[29] National Center for Biotechnology Information. PubChem compound summary for CID 448042, Y-27632 dihydrochloride. 2022. https://pubchem.ncbi.nlm.nih.gov/compound/Fasudil#section=2D-Structure. Accessed 10 Sep 2022.

[30] Xi Y, Xu PF (2021). Therapeutic potentials of fasudil in liver fibrosis. World J Gastroenterol.

[31] Hirsova P, Ibrahim SH, Krishnan A, Verma VK, Bronk SF, Werneburg NW (2016). Lipid-induced Signaling causes release of inflammatory extracellular vesicles from hepatocytes. Gastroenterology.

[32] Shibuya M, Suzuki Y, Sugita K, Saito I, Sasaki T, Takakura K (1992). Effect of AT877 on cerebral vasospasm after aneurysmal subarachnoid haemorrhage: results of a prospective placebo-controlled double-blind trial. J Neurosurg.

[33] Jacobs M, Hayakawa K, Swenson L, Bellon S, Fleming M, Taslimi P (2006). The structure of dimeric ROCK I reveals the mechanism for ligand selectivity. J Biol Chem.

[34] Sasaki Y, Suzuki M, Hidaka H (2002). The novel and specific Rho-kinase inhibitor (S)-(+)-2-methyl-1-[(4-methyl-5-isoquinoline)sulfonyl]-homopiperazine as a probing molecule for Rho-kinase-involved pathway. Pharmacol Ther.

[35] Hobson AD, Judge RA, Aguirre AL, Brown BS, Cui Y, Ding P (2018). Identification of selective dual ROCK1 and ROCK2 inhibitors using structure-based drug design. J Med Chem.

[36] Shen M, Zhou S, Li Y, Pan P, Zhang L, Hou T (2013). Discovery and optimization of triazine derivatives as ROCK1 inhibitors: molecular docking, molecular dynamics simulations and free energy calculations. Mol Biosyst.

[37] National Center for Biotechnology Information. PubChem compound summary for CID 3547, Fasudil. Reactive red 120. 2022. https://pubchem.ncbi.nlm.nih.gov/compound/Y-27632-dihydrochloride#section=2D-Structure. Accessed 10 Sep 2022.

[38] National Center for Biotechnology Information. PubChem compound summary for CID 3064778, Hydroxyfasudil. https://pubchem.ncbi.nlm.nih.gov/compound/Hydroxyfasudil. Accessed 28 Dec 2022.

[39] National Center for Biotechnology Information. PubChem compound summary for CID 448043, H-1152P. https://pubchem.ncbi.nlm.nih.gov/compound/448043. Accessed 28 Dec 2022.

[40] Matsukawa J, Matsuzawa A, Takeda K, Ichijo H (2004). The ASK1-MAP kinase cascades in mammalian stress response. J Biochem.

[41] Wang Y, Wen H, Fu J, Cai L, Li PL, Zhao CL (2020). Hepatocyte TNF receptor-associated factor 6 aggravates hepatic inflammation and fibrosis by promoting lysine 6–linked polyubiquitination of apoptosis signal-regulating kinase 1. Hepatology.

[42] Brys R, Gibson K, Poljak T, van der Plas S, Amantini D (2020). Discovery and development of ASK1 inhibitors. Prog Med Chem.

[43] Loomba R, Lawitz E, Mantry PS, Jayakumar S, Caldwell SH, Arnold H (2018). The ASK1 inhibitor selonsertib in patients with nonalcoholic steatohepatitis: a randomized, phase 2 trial. Hepatology.

[44] Harrison SA, Wong VW-S, Okanoue T, Bzowej N, Vuppalanchi R, Younes Z (2019). Safety and efficacy of selonsertib for the treatment of bridging fibrosis or compensated cirrhosis due to nonalcoholic steatohepatitis (NASH): results of the phase 3 stellar studies. Hepatology.

[45] Gibson TS, Johnson B, Fanjul A, Halkowycz P, Dougan DR, Cole D (2017). Structure-based drug design of novel ASK1 inhibitors using an integrated lead optimization strategy. Bioorg Med Chem Lett.

[46] Lovering F, Morgan P, Allais C, Aulabaugh A, Brodfuehrer J, Chang J (2018). Rational approach to highly potent and selective apoptosis signal-regulating kinase 1 (ASK1) inhibitors. Eur J Med Chem.

[47] Lanier M, Pickens J, Bigi SV, Bradshaw-Pierce EL, Chambers A, Cheruvallath ZS (2017). Structure-based design of ASK1 inhibitors as potential agents for heart failure. ACS Med chem lett.

[48] Bunkoczi G, Salah E, Filippakopoulos P, Fedorov O, Müller S, Sobott F (2007). Structural and functional characterization of the human protein kinase ASK1. Structure.

[49] National Center for Biotechnology Information. PubChem compound summary for CID 71245288, Selonsertib. 2022. https://pubchem.ncbi.nlm.nih.gov/compound/Selonsertib#section=2D-Structure. Accessed 10 Sep 2022.

[50] Garuti L, Roberti M, Bottegoni G (2015). Multi-kinase inhibitors. Curr Med Chem.

[51] Broekman F (2011). Tyrosine kinase inhibitors: multi-targeted or single-targeted?. World J Clin Oncol.

[52] Irwin JJ, Shoichet BK (2005). ZINC - A free database of commercially available compounds for virtual screening. J Chem Inf Model.

[53] Hu Z, Wang C, Glunz PW, Li J, Cheadle NL, Chen AY (2020). Discovery of a phenylpyrazole amide ROCK inhibitor as a tool molecule for in vivo studies. Bioorg Med Chem Lett.

[54] Green J, Cao J, Bandarage UK, Gao H, Court J, Marhefka C (2015). Design, synthesis, and structure-activity relationships of pyridine-based rho kinase (ROCK) inhibitors. J Med Chem.

[55] Shaw D, Hollingworth G, Soldermann N, Sprague E, Schuler W, Vangrevelinghe E (2014). Novel ROCK inhibitors for the treatment of pulmonary arterial hypertension. Bioorg Med Chem Lett.

[56] Xin Z, Himmelbauer MK, Jones JH, Enyedy I, Gilfillan R, Hesson T (2020). Discovery of CNS-penetrant apoptosis signal-regulating kinase 1 (ASK1) inhibitors. ACS Med Chem Lett.

[57] Vogel SM, Bauer MR, Boeckler FM (2011). DEKOIS: demanding evaluation kits for objective in silico screening—a versatile tool for benchmarking docking programs and scoring functions. J Chem Inf Model.

[58] Himmelbauer MK, Xin Z, Jones JH, Enyedy I, King K, Marcotte DJ (2019). Rational design and optimization of a novel class of macrocyclic apoptosis signal-regulating kinase 1 inhibitors. J Med Chem.

[59] Terao Y, Suzuki H, Yoshikawa M, Yashiro H, Takekawa S, Fujitani Y (2012). Design and biological evaluation of imidazo[1,2-a]pyridines as novel and potent ASK1 inhibitors. Bioorg Med Chem Lett.

[60] Liles JT, Corkey BK, Notte GT, Budas GR, Lansdon EB, Hinojosa-Kirschenbaum F (2018). ASK1 contributes to fibrosis and dysfunction in models of kidney disease. J Clin Invest.

[61] Mysinger MM, Carchia M, Irwin JJ, Shoichet BK (2012). Directory of useful decoys, enhanced (DUD-E): better ligands and decoys for better benchmarking. J Med Chem.

[62] el Kerdawy AM, Osman AA, Zaater MA (2019). Receptor-based pharmacophore modeling, virtual screening, and molecular docking studies for the discovery of novel GSK-3β inhibitors. J Mol Model.

[63] Singh O, Shillings A, Craggs P, Wall I, Rowland P, Skarzynski T (2013). Crystal structures of ASK1-inhibtor complexes provide a platform for structure-based drug design. Protein Sci.

[64] Chang E. Apoptosis signal-regulating kinase 1 inhibitors, WO2011097079A1. (2011)

[65] Koes DR, Camacho CJ (2012). ZINCPharmer: pharmacophore search of the ZINC database. Nucleic Acids Res.

[66] Irwin JJ, Sterling T, Mysinger MM, Bolstad ES, Coleman RG (2012). ZINC: A free tool to discover chemistry for biology. J Chem Inf Model.

[67] Oprea TI (2000). Property distribution of drug-related chemical databases. J Comput Aided Mol Des.

[68] Kazius J, McGuire R, Bursi R (2005). Derivation and validation of toxicophores for mutagenicity prediction. J Med Chem.

[69] Veber DF, Johnson SR, Cheng HY, Smith BR, Ward KW, Kopple KD (2002). Molecular properties that influence the oral bioavailability of drug candidates. J Med Chem.

[70] Baell JB, Holloway GA (2010). New substructure filters for removal of pan assay interference compounds (PAINS) from screening libraries and for their exclusion in bioassays. J Med Chem.

[71] Baell J, Walters MA (2014). Chemistry: chemical con artists foil drug discovery. Nature.

[72] Daina A, Michielin O, Zoete V (2017). SwissADME: a free web tool to evaluate pharmacokinetics, drug-likeness and medicinal chemistry friendliness of small molecules. Sci Rep.

[73] Daina A, Michielin O, Zoete V (2014). ILOGP: a simple, robust, and efficient description of n-octanol/water partition coefficient for drug design using the GB/SA approach. J Chem Inf Model.

[74] Daina A, Zoete V (2016). A BOILED-Egg to predict gastrointestinal absorption and brain penetration of small molecules. ChemMedChem.

[75] Finch A, Pillans P (2014). P-glycoprotein and its role in drug-drug interactions. Aust Prescr.

[76] Martin YC (2005). A bioavailability score. J Med Chem.

[77] Sander T, Freyss J, von Korff M, Rufener C (2015). DataWarrior: an open-source program for chemistry aware data visualization and analysis. J Chem Inf Model.

[78] Houston DR, Yen LH, Pettit S, Walkinshaw MD (2015). Structure- and ligand-based virtual screening identifies new scaffolds for inhibitors of the oncoprotein MDM2. PLoS ONE.

[79] Abraham MJ, Murtola T, Schulz R, Páll S, Smith JC, Hess B (2015). GROMACS: High performance molecular simulations through multi-level parallelism from laptops to supercomputers. SoftwareX.

[80] Lindorff-Larsen K, Piana S, Palmo K, Maragakis P, Klepeis JL, Dror RO (2010). Improved side-chain torsion potentials for the Amber ff99SB protein force field. Proteins.

[81] Wang J, Wolf RM, Caldwell JW, Kollman PA, Case DA (2004). Development and testing of a general amber force field. J Comput Chem.

[82] Wang J, Wang W, Kollman PA, Case DA (2006). Automatic atom type and bond type perception in molecular mechanical calculations. J Mol Graph Model.

[83] Sousa Da Silva AW, Vranken WF (2012). ACPYPE—AnteChamber PYthon parser interfacE. BMC Res Notes.

[84] Bussi G, Donadio D, Parrinello M (2007). Canonical sampling through velocity rescaling. J Chem Phys.

[85] Parrinello M, Rahman A (1981). Polymorphic transitions in single crystals: a new molecular dynamics method. J Appl Phys.

[86] Hess B, Bekker H, Berendsen HJC, Fraaije JGEM (1997). LINCS: a linear constraint solver for molecular simulations. J Comput Chem.

[87] Darden T, York D, Pedersen L (1993). Particle mesh Ewald: an N⋅log(N) method for Ewald sums in large systems. J Chem Phys.

[88] Pettersen EF, Goddard TD, Huang CC, Couch GS, Greenblatt DM, Meng EC (2004). UCSF Chimera–a visualization system for exploratory research and analysis. J Comput Chem.

