# Supplementary material for: Discovery of dual rho-associated protein kinase 1 (ROCK1)/apoptosis signal–regulating kinase 1 (ASK1) inhibitors as a novel approach for non-alcoholic steatohepatitis (NASH) treatment
Source: BMC Chem. 2024 Jan 3;18(1):2. doi: 10.1186/s13065-023-01081-3 (PMC10765837; doi:10.1186/s13065-023-01081-3)
Supplement: Supplementary file 1 — Additional file 1. S1. Selection of the X-ray crystallographic structures for ROCK1 and ASK1 and training set generation. S2. Assessment metrics used to assess the performance of the constructed pharmacophore models. S3. The quantitative and qualitative description of the generated Pharmacophore models. S4. Self-docking validation of the used molecular docking protocol. S5. Common binding pattern molecules mapped onto the chosen pharmacophore model. [file 13065_2023_1081_MOESM1_ESM.pdf]

Supporting Information

Discovery of Dual Rho-Associated Protein Kinase 1 (ROCK1)/Apoptosis Signal–Regulating Kinase 1 (ASK1) Inhibitors for the Treatment of Non-Alcoholic Steatohepatitis (NASH)

Yara A. Zaky[1], Mai W. Rashad[1], Marwa A. Zaater[2] and Ahmed M. El Kerdawy[1,3,4]*

[1]*Department of Chemistry, School of Pharmacy, Newgiza University (NGU), Newgiza, km 22 Cairo-Alexandria Desert Road, Cairo, Egypt.*

[2]*Master Postgraduate Program, Department of Pharmaceutical Chemistry, Faculty of Pharmacy, Cairo University, Cairo, Egypt.*

[3]*Department of Pharmaceutical Chemistry, Faculty of Pharmacy, Cairo University, Cairo, Egypt.*

[4]*School of Pharmacy, College of Science, University of Lincoln, Joseph Banks Laboratories, Green Lane, Lincoln, Lincolnshire, United Kingdom.*

Table of Content

S1. Selection of the X-ray crystallographic structures for ROCK1 and ASK1 and training set generation…………………………………………………………………………………...2

S2. Assessment metrics used to assess the performance of the constructed pharmacophore models………………………………………………………………………………………5

S3. The quantitative and qualitative description of the generated Pharmacophore models…….6

S4. Self-docking validation of the used molecular docking protocol…………………………..7

S5. Common binding pattern molecules mapped onto the chosen pharmacophore model …………………………..9

S1. Selection of the X-ray crystallographic structures for ROCK1 and ASK1 and training set generation

Co-crystalized ROCK1 inhibitors interact mainly at three region: the hinge region, the catalytic residue Lys105, and gate area. They interact with the hinge region and the catalytic residue Lys105 via classical hydrogen bonds and hydrophobic interactions. Interactions with Asp216 of the DFG-motif at the gate area were also observed in very few structures. Focusing on the hinge region, interactions frequently occur with Tyr155 and Met156 and to a lesser extent with Gln154. Most inhibitors also interact with the catalytic residue, Lys105. Moreover, the inhibitors are involved in hydrophobic interactions with the hydrophobic residues Leu82 and Val90 as well.

ASK1 inhibitors show a similar binding pattern interacting with the hinge region and the catalytic residue Lys709 via classical hydrogen bonds and hydrophobic interactions. Interactions with Asp822 of the DFG-motif at the gate area were observed in one inhibitor only. At the hinge region, ASK1 inhibitors usually interact with Gly756 and Val757 and less frequently with Met754 and Gln755. All inhibitors interacted with the catalytic Lys709. Furthermore, most of the inhibitors interacted with Leu686.

Afterward, the inhibitors were grouped according to their observed interactions after their analysis individually. Overall, ROCK1 inhibitors of the selected group interact through hydrogen bonds with Tyr155 and Met156 (at the hinge region), Lys105 (catalytic residue), and hydrophobic interactions through aromatic rings. On the other hand, ASK1 selected inhibitors interacted with the kinase domain through hydrogen bond interactions at Val757 (hinge region), Lys709 (catalytic residue), and various hydrophobic interactions at Leu686.

The co-crystallized inhibitors in the chosen ROCK1 and ASK1 protein structures constituting the training set include the following compounds:

ROCK1 inhibitors:

1) VFS (PDB ID: 7JOU) was discovered during a structure-activity relationship optimization of some phenylpyrazole amides to identify potential dual ROCK1 and ROCK2 inhibitors. It exhibited a high potency with IC_{50} values of 7.9 nM and 2.3 nM on ROCK1 and ROCK2, respectively. VFS binds in a similar pattern at both ROCK1 and ROCK2 which is seen among other ROCK1 and ROCK2 crystal structures. In ROCK1, VFS binds to the hinge region via hydrogen bonding with Tyr155 and Met156. Moreover, the amide carbonyl group interacts

with the conserved Lys105 via a hydrogen bond with the terminal phenyl ring directed under the p-loop.

2) 4KH (PDB ID: 4YVC) was discovered during a study aimed to design and establish the structure-activity relationship of pyridine-based ROCK inhibitors. The whole study was based on this compound which was identified through high-throughput screening. It was chosen due to its potential to be developed further producing more potent and selective inhibitors. It exhibited K_i of 1.1 μM more potent than against the off-target protein kinase A (K_i > 5 μM) which is a member of the AGC family as well. 4KH pyridine group interacts within the hinge region via a hydrogen bond with Met156 and its amide carbonyl is within a hydrogen bonding distance with the catalytic Lys105.

3) 3J7 (PDB ID: 4W7P) was discovered through structure-based drug design and was tested through pharmacokinetic/pharmacodynamic experiments to identify potential treatment options for pulmonary arterial hypertension through targeting ROCK1. 3J7 has an IC_{50} of 32 nM and binds to ROCK1 hinge region Met156 located via its indazole ring, moreover, it binds to the catalytic Lys105 via a nitrogen atom at its pyridopyrimidine core. It also achieves an interesting hydrogen bond interaction with Asp160.

ASK1 inhibitors:

1) RFG (PDB ID: 6VRE) was discovered through medicinal chemistry studies that aimed to discover potent and CNS-penetrating ASK1 inhibitors. Due to its involvement in apoptosis and inflammation, ASK1 is believed to have a therapeutic value in neurological diseases. RFG has an IC50 of 138 nM and binds to ASK1 hinge region Val757 via an amide carbonyl oxygen and to its catalytic Lys709 by its triazole nitrogen.

2) 8V7 (PDB ID: 5V24) was discovered in a study that aimed to integrate several lead generation and optimization techniques to design novel and potent ASK1 inhibitors. It has an IC_{50} of 400 nM and binds to Val757 of the hinge region through its amide carbonyl. Through its triazole ring, 8V7 binds to the hinge region Met754 and the catalytic Lys709 via hydrogen bonding.

3) 9E1 (PDB ID: 5VIL) was discovered during efforts to further optimize potent ASK1 inhibitors to increase potency and selectivity and improve physicochemical properties. This inhibitor has a high potency (IC_{50} = 1.5 nM) and binds to ASK1 hinge region Val757 through

its amide carbonyl oxygen. Moreover, it interacts with Gly756 through a hydrogen bond with its sulphonamide. Through its triazole group, it interacts with the catalytic Lys709.

4) 8GY (PDB ID: 5UOX) was discovered by utilizing structure-based drug design, deconstruction, and re-optimization of an already known ASK1 inhibitor. When tested, this compound displayed efficient MAP3K pathway inhibition. Furthermore, when investigated in a heart model of cardiac injury, it reduced the size of the infarcted tissue. 8GY has a significantly high potency with an pIC_{50} of 8.3±0.06. Through its amide carbonyl, it interacts with Gly756 and Val757 at the hinge region and by its triazole group, it interacts with hinge region Met754 and catalytic loop Lys709. Finally, by its hydroxyl group, it interacts with Asn808 and Ser821.

S2. Assessment metrics used to assess the performance of the constructed pharmacophore models.

Table S1. *Pharmacophore models performance assessment metrics*

Assessment Metric	Equation	Insight
Sensitivity (Se)	$Se = \frac{TP}{A}$	The ability of the model to identify truly active molecules as hits.
Specificity (Sp)	$Sp = \frac{TN}{N - A}$	The ability of the model to identify decoys and discard them.
Yield of Actives (Ya)	$Ya = \frac{TP}{n}$	The pharmacophore model hit rate.
Enrichment (E)	$E = \frac{TP/n}{A/N}$	How well the pharmacophore model performs better than randomness (random selection in retrieving active compounds).
Accuracy (Acc)	$acc = \frac{TP + TN}{N}$	The accuracy of the pharmacophore in differentiating between actives and inactive compounds.
Discrimination Ratio (DR)	$DR = \frac{Se}{Sp}$	Measures the predictive accuracy of the pharmacophore model when screening active and inactive compounds.
F1 score (f1)	$f1 = \frac{TP}{TP + \frac{1}{2}(FP + FN)}$	Measures the overall quality of the model in differentiating between active and inactive compounds.
Mathew's Correlation Coefficient (MCC)	$MCC = \frac{(TPxTN) - (FPxFN)}{\sqrt{(TP + FN)(TP + FP)(TN + FP)(TN + FN)}}$	Generates a high score only if the pharmacophore model can predict the majority of actives and inactive compounds occurrences.

TP is the number of true positive, **TN** is the number of true negative, **FP** is the number of false positive, **FN** is the number of false negative, **A** is the number of actives, **n** is the number of selected compounds as hits and **N** is the total number of compounds in the dataset.

S3. The quantitative and qualitative description of the generated Pharmacophore models

Table S2. *The quantitative and qualitative description of the generated Pharmacophore models' feature*

PHARMACOPHORE MODEL NO.	NO. OF FEATURES	FEATURE TYPES						NO. OF EXCLUDED VOLUMES	
		F1	F2	F3	F4	F5	Partial match	Automatic	Manual
**1**	4	Acc	Aro	Acc	Acc2	-	-	-	44
**2**	3	Acc	Aro	Acc	-	-	-	Auto 4W7P (1.0**)	-
**3**	4	Acc	Aro	Aro	Acc	-	-	Auto 4W7P (1.2**)	-
**4**	3	Aro	Acc	Acc	-	-	-	Auto 4W7P (1.6**)	-
**5**	4	Acc	Acc	Acc	Aro	-	-	-	-
**6**	4	Acc	Aro	Acc	Don	-	-	-	-
**7**	4	Aro	Acc	Acc	Acc	-	-	Auto 4W7P (0.5**)	-
**8**	5	Acc	Acc	Acc	Aro	Aro	At least 1 (F2, F3)*	Auto 4W7P (0.7**)	-
**9**	4	Acc	Aro	Acc	Acc	-	At least 1 (F3, F4)*	Auto 4W7P (0.8**)	120
**10**	5	Acc	Aro	Aro	Acc	Acc	At least 1 (F4, F5)*	-	-
**11**	5	Acc	Acc	Acc	Aro	Aro	At least 1 (F2, F3)*	Auto 4W7P (0.8**)	103
**12**	5	Acc	Acc	Aro	Acc	Aro	At least 1 (F1, F2)*	Auto 4W7P (1.0**)	100
**13**	4	Aro	Acc	Acc	Acc	-	At least 1 (F3, F4)*	Auto 4W7P (0.5**)	200

* Two acceptor features located near the hinge region to describe residue position discrepancy in binding sites of ROCK1 and ASK1 (ROCK1: Tyr155 & Met156 and ASK1: Glu756 & Val757).

** Radius of excluded volume in Å.

S4. Self-docking validation of the used molecular docking protocol

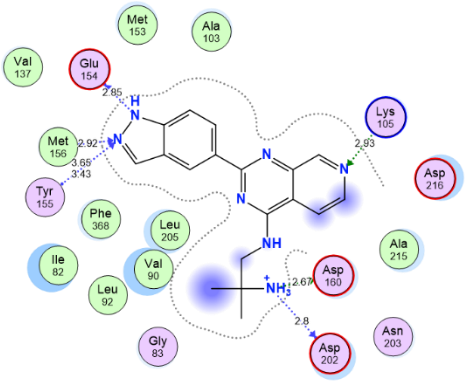

(a)

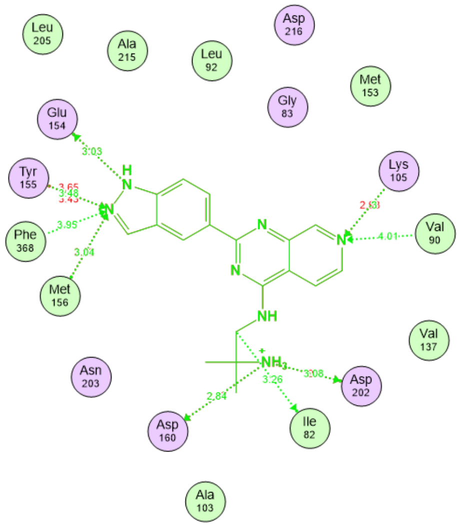

(b)

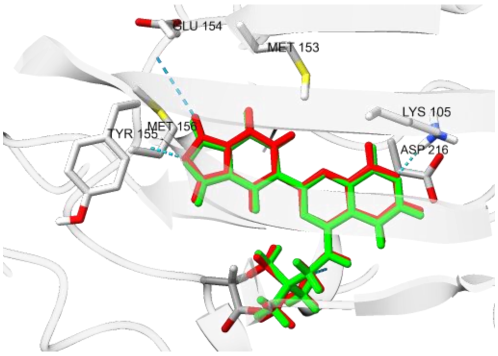

(c)

Figure S1. *2D ligand interactions diagram of the co-crystalized ligand (a) and 2D diagram (b) and 3D representation (c) of the overlay of the self-docking pose (green) and co-crystalized ligand (red) in the ROCK1 binding site (PDB ID: 4W7P).*

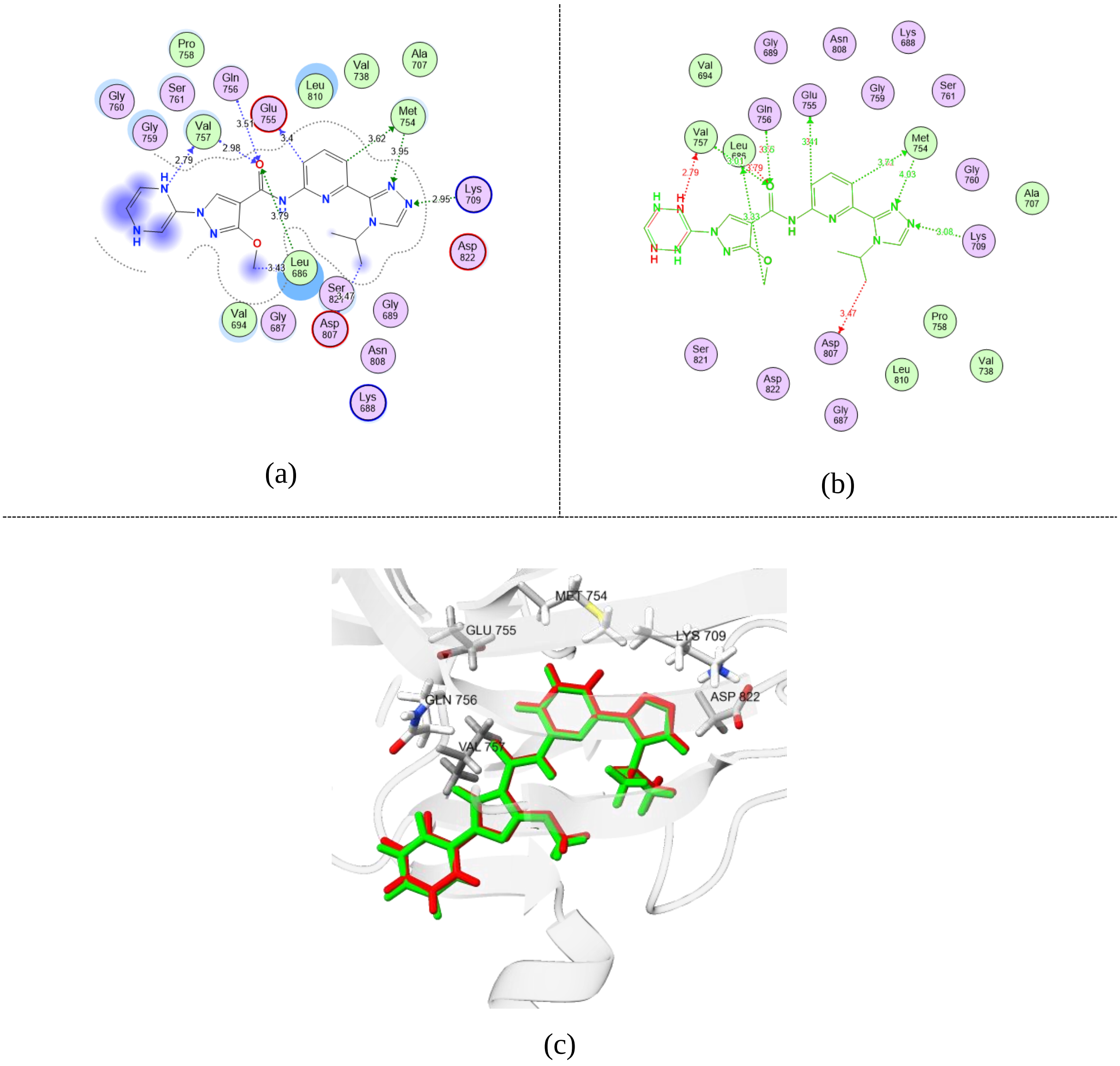

Figure S2. *2D ligand interactions diagram of the co-crystalized ligand (a) and 2D diagram (b) and 3D representation (c) of the overlay of the self-docking pose (green) and co-crystalized ligand (red) in the ASK1 binding site (PDB ID: 6VRE).*

S5. Common binding pattern molecules mapped onto the chosen pharmacophore model.

ZINC ID	Pharmacophore Map
ZINC04968098	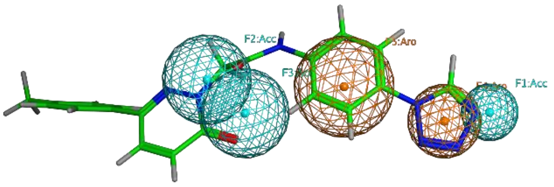
ZINC48234390	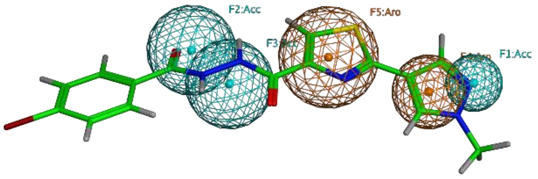
ZINC12428844	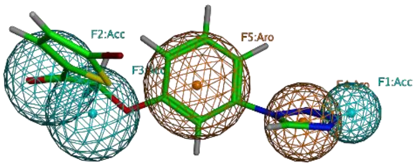
ZINC89779407	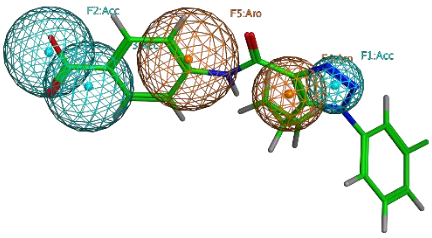
ZINC20446787	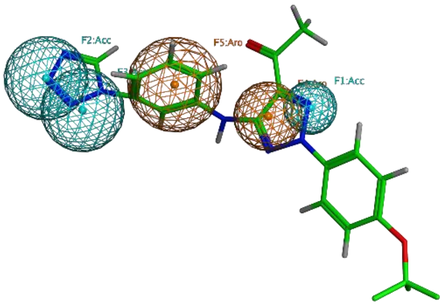
ZINC40129807	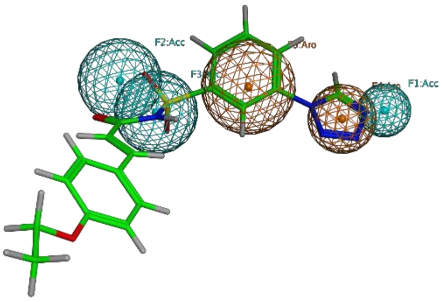

ZINC32960731	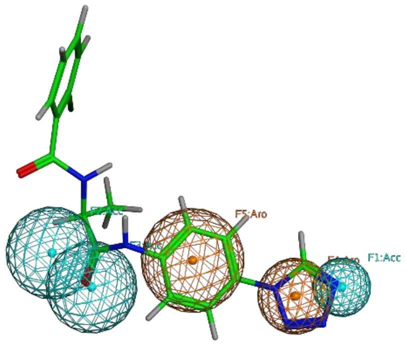
ZINC73988575	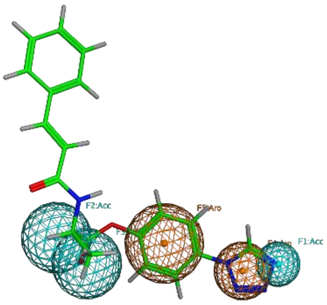
ZINC72263787	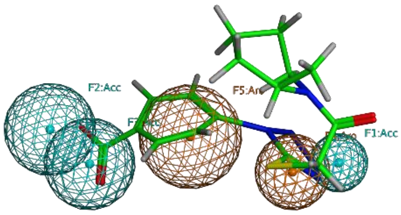
ZINC89638949	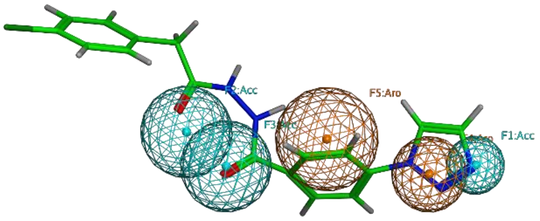
ZINC05938451	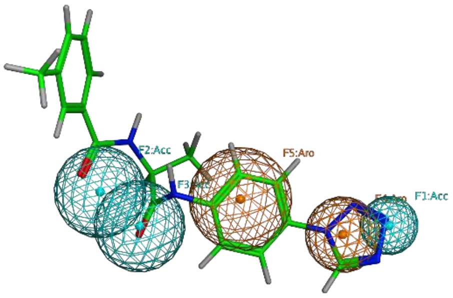

ZINC45947713	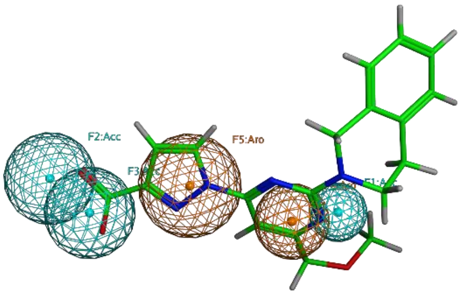
ZINC66266481	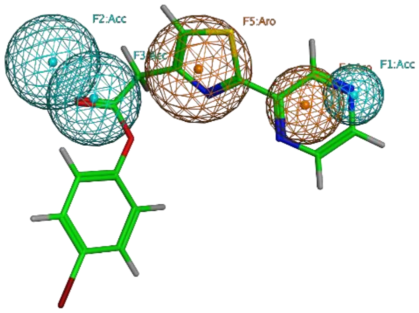
ZINC00637673	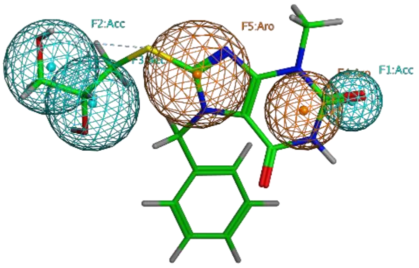
ZINC77167529	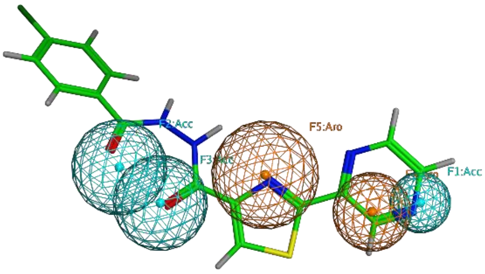
ZINC90394330	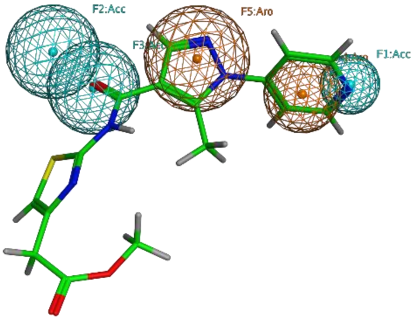

ZINC60499106	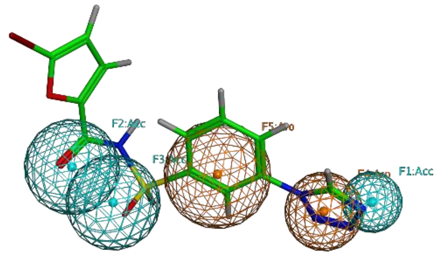
ZINC20978296	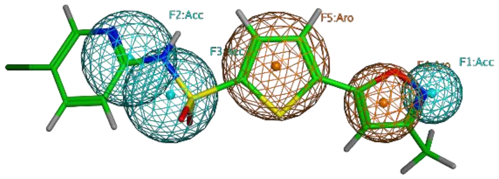
ZINC72465685	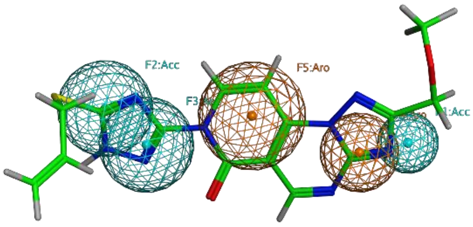
ZINC36693793	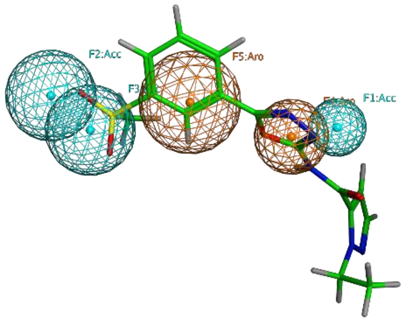
ZINC78082037	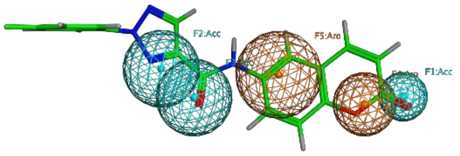
ZINC36693728	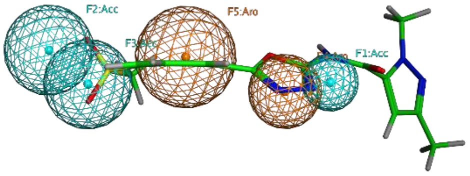
ZINC04968107	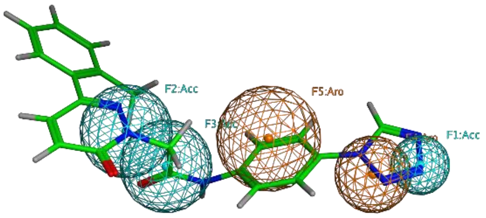

ZINC69582168	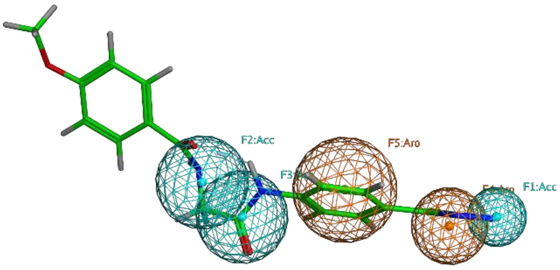
ZINC69582176	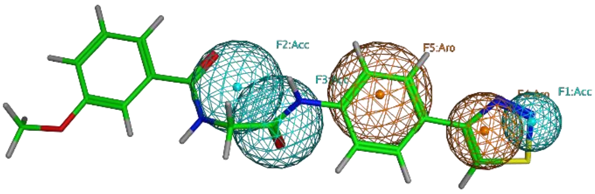
ZINC09503410	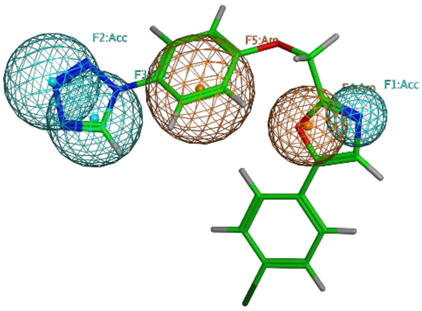
ZINC29463219	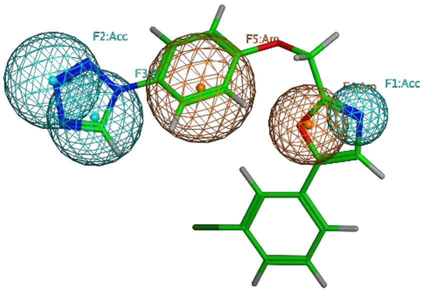
ZINC91041750	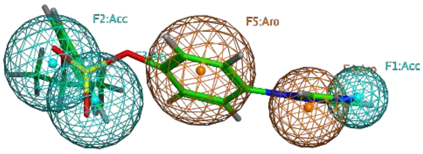
ZINC56905939	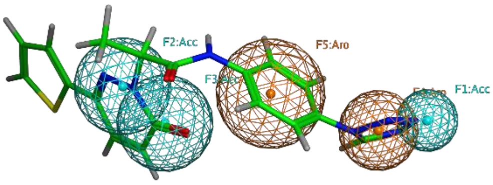

ZINC21466280	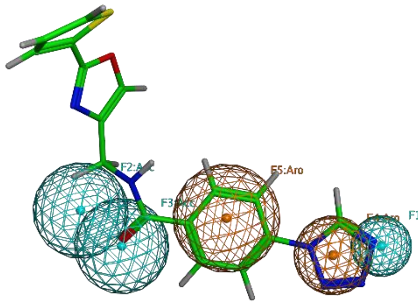
ZINC77943297	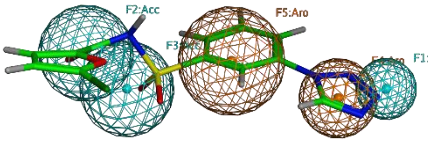
ZINC71715331	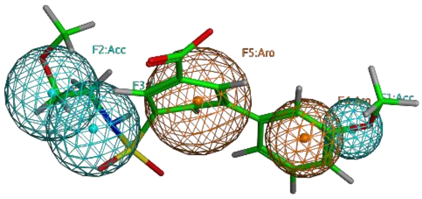
ZINC04088663	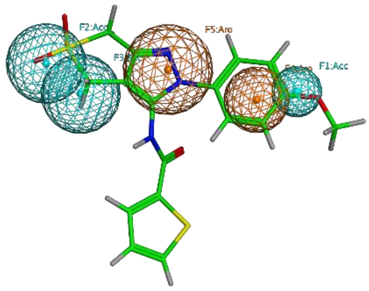
ZINC49028294	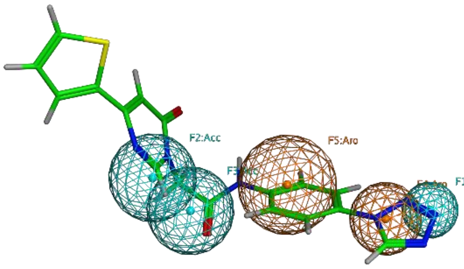
ZINC72014041	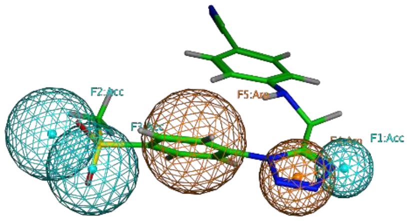
ZINC90422635	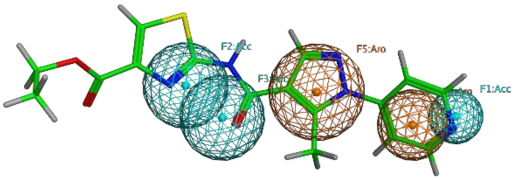

ZINC64802850	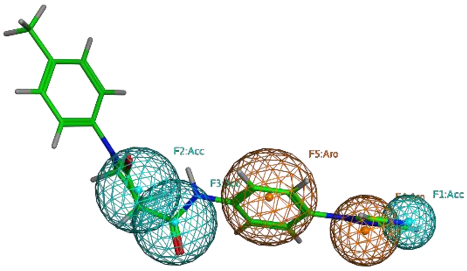
ZINC50059342	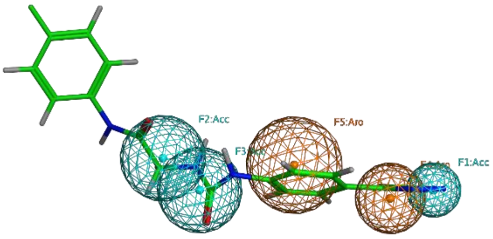
ZINC91701857	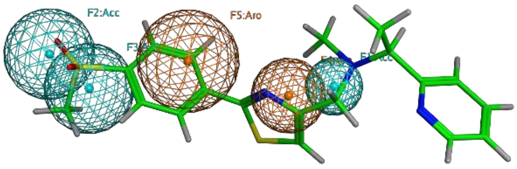
ZINC58364038	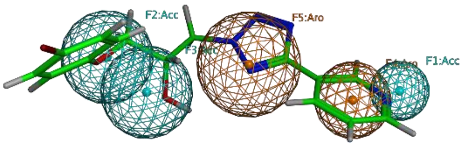
